# Impact of pesticides exposure on *Archachatina marginata* snails in four Cameroon monomodal rainforest sites

**DOI:** 10.1371/journal.pone.0297369

**Published:** 2024-03-04

**Authors:** Annick Niquaise Enangue Njembele, Maeva Arielle Patience Seppo Njembele, Emmanuel Henoch Dicka kwambe, Alexis Hamdja Ngoniri, Sylvie Ntyam epse Ondo, Kingsley Agbor Etchu

**Affiliations:** 1 Station Specialized on Marine Ecosystem, Institute of Agricultural Research for Development (IRAD), Cameroon; 2 Master in Coastal and Marine Integrated Environment, Institute of Fisheries and Aquatic Sciences (ISH), University of Douala, Douala, Cameroun; 3 National Institute of Cartography (INC), Yaoundé, Cameroon; 4 Nkolbisson Headquarter, Institute of Agricultural Research for Development (IRAD), Nkolbisson, Yaoundé, Cameroon; University of Ibadan, NIGERIA

## Abstract

Cameroon monomodal rainforest zone has a strong agricultural activity and is therefore exposed to pesticides. Furthermore, the area possesses climatic factors that favor the growth of *Achatinadea* snails known as African giant snails, a delicacy for the local population. The present study aimed to evaluate pesticides contamination (less vs more exposed areas) through assessment of exposure and impact on *Achatinadea* snails. *Achatinadea* snails were collected within intensive agricultural areas (Njombe and Kribi rural) and in areas with less agricultural activity (Ebodje and Dibombari). Collection was performed at night between July and September 2020 using an adapted square kilometer method. Type, number, weight, and size of the collected snails were analyzed and compared using Welsh’s One-way Analysis of variance (ANOVA). After removing the soft part from the shell, the presence of pesticides was determined using mass spectrometry. Histological analysis of kidney and ovo-testis was performed using eosin-hematoxylin staining. Results showed that the main variety of snails collected are *Archachatina marginata*. In areas with less agricultural activity, snails are bigger than those from more agricultural areas heavily using pesticides. Furthermore, pesticides detection showed that glyphosate, but not metalaxyl, is present in animals coming from all the collection sites. Cypermethrin was found in all the samples except in those from Dibombari. Histology revealed that the structure of the kidney and ovo-testis of snails from more exposed areas is impaired. In conclusion, this study revealed that some pesticides are transferred to snail and impair the structure of important organs.

## Introduction

Cameroon is a country situated in Central Africa. It shares a large border in the north and west with Nigeria. Cameroon economy is mostly based on agriculture. Due to the climate, the country is divided into five agro-ecological zones (Sudano-Sahelian, Guinean High Savannah, Western Highland Plateau, Bimodal Rainforest and Monomodal Rainfores**t**). The Monomodal Rainforest zone is known to have a strong agricultural activity in the country. Several industrial plantations such as CDC (Cameroon Development Corporation) and SOCAPALM (Cameroon oil palm company), PHP (Plantation Haut Penja) are found in this area. The local population are in majority farmers and they cultivate several crops for their consumption and to supply the urban area. In order to increase their productivity, farmers have turn to the use of pesticides [1–4], which use has increased the agricultural production in the area [4]. However, the best practice or safe use of pesticides is not yet well known by the majority of farmers, which has led to environmental biohazard [1, 3, 5–10]. Consequently, the area is highly prone to pesticides contamination. Additionally, the area is humid and favor the growth of land snails. Land snails are also attracted to the type of culture that are cultivated in the area such as palm oil nuts, bananas, and papaya trees, tomatoes, leafy vegetables, and diverse tuber and root plants such as cassava and sweet potatoes [11]. Snails are feeding on the fruits and leaves found on the ground of the farms [11]. The common edible snail group in the area is *Achatinadea* group also known as African giant snail. *Achatinadea* is the largest snail variety living in the soil. They are nocturnal and hermaphrodite (possess both types of reproductive organs) [12–14]. Among this group are found 3 species in Cameroon: *Achatina achatina*, *Achatina fulica* and *Archachatina marginata* [11, 14]. Various studies have shown that *Achatinadea* snail meat possesses many nutritional benefits [15–18] and is very appreciated by the local population [19]. Several studies have shown that pesticides and heavy metals are transferred to snails (land and freshwater snails) [20–24]. The land snail species that was mostly used as biomarker to monitor pesticides and heavy metals is *Helix aspersa* [21, 25, 26]. Most of the studies carried out on African giant snails are on their farming and nutritional aspect [15, 16, 27–35]. There are only a few studies on their potential biohazard contamination to human [33, 36–39]. The present study aims to evaluate pesticide contamination and the impact on *Achatinadae* snails in Cameroon agricultural areas. Our hypothesis is that *Achatinadae* snails could be contaminated and impaired by pesticides since they are living on soils of pesticide-exposed farms.

## Materials and methods

### Description of the study area

**Fig 1:**
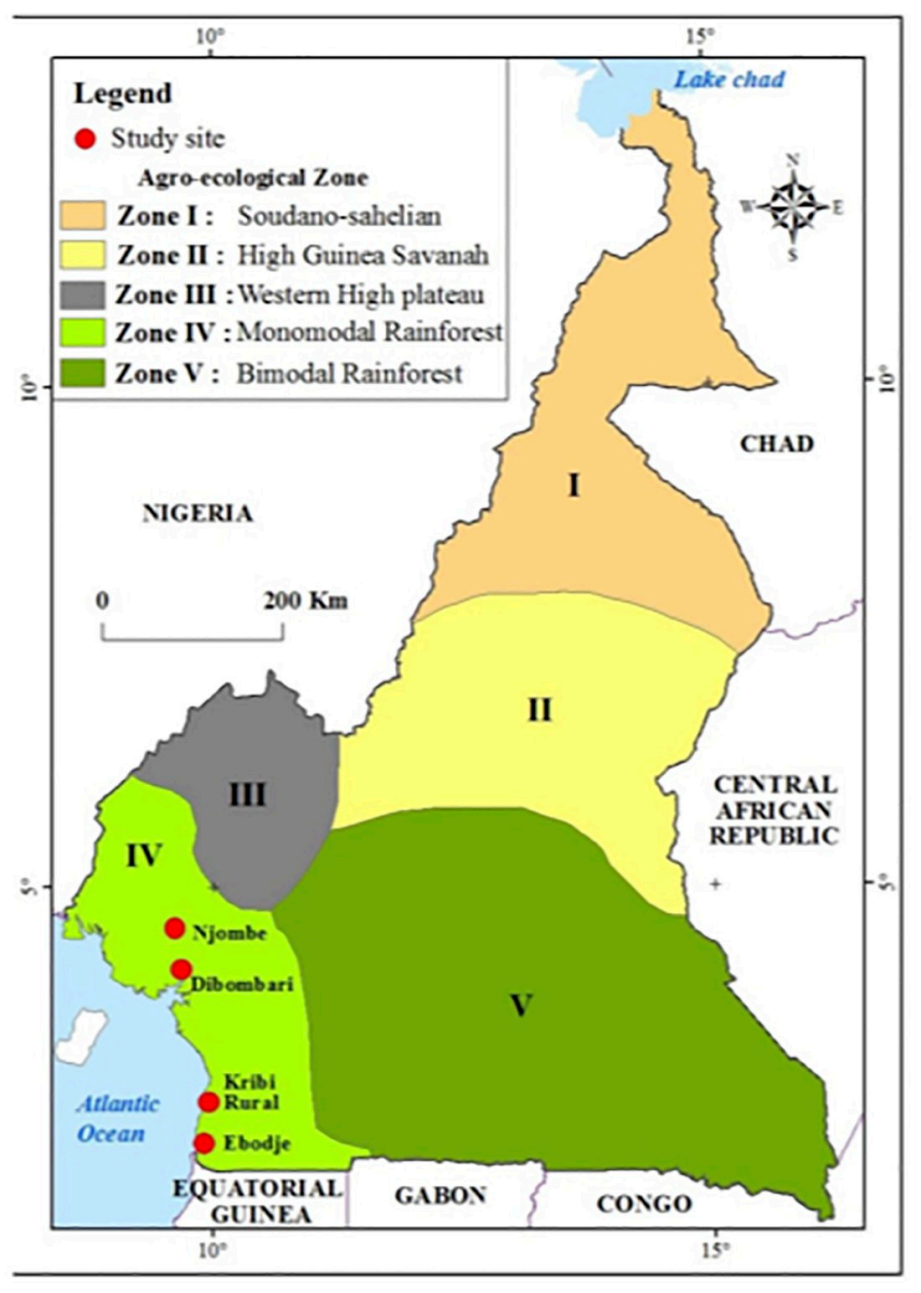
Map of the study area: Cameroon is divided into five agro-ecological zone. The study was performed within the Zone IV (in light green). Coordinates of the four collection areas coordinates were taken using a GPS and localization placed in Cameroon map showed here with red dots.

The study was performed within two regions: the South within the Ocean division (kribi rural and Ebodje), and the Littoral within Moungo division (Dibombari and Njombe) (Fig 1). According to the agroecological division of Cameroon, the study sites belong to the humid forest zone with mono-modal rainfall (consisting of one rainy season followed by one dry season). The Moungo division is characterized by the presence of volcanoes such as Mounts Manengouba and Koupe. The combination of all of these factors offers favorable climatic conditions for agriculture and also for the reproduction and development of *Achatinadea* snails. Ocean division is the nearest division to the Atlantic Ocean and the Congo Basin, that gives also the area a microclimate suitable for agriculture. More, within these two divisions are found several agricultural industries such as CDC, PHP, SOCAPALM that uses for some pesticides airplanes sprayer (done twice a week, CDC and PHP) and manual for SOCAPALM. These areas have small population. According to the 2022 census of the Cameroon National Institute of Statistic, these areas comprise 8,890 inhabitants for Njombe, 55,401 inhabitants for Kribi, 17,141 inhabitants for Dibombari and 6,000 inhabitants for Campo where Ebodje is a village. Local population is mostly farmers that cultivate food to supply major cities Douala and Yaoundé.

### Sample collection

*Achatinadea* samples were collected into two types of sites within the monomodal rainfores**t** area: intensive agricultural areas (Njombe and Kribi rural) versus area with less agricultural activity (Ebodje and the wild forest of Dibombari). Collection was performed at night between July to September 2020 using an adaptation of a square kilometer method [40, 41]. Coordinates of the collection areas were also taken using a GPS Triton 200.

### Analysis of the general structure of snails

Collected snails of each group were counted as well as their weight was taken using sensible scale. General aspect: shell condition and the soft part (head and foot) were also analyzed.

### Histology

Four snails from each collected groups were randomly chosen for histological studies. Snails were firstly left without food for 48 h to let them to digest all the food in their body. Animals were euthanized into boiled water and directly put on the fixative solution formaldehyde 4%. Dissection was done according to the protocol of Lőw *et al*. [42]. The soft part was removed from their shell then, ovo-testis and kidney were excised out. Furthermore, graded dehydration of the excised organs was done using 70% to 100% ethyl alcohol in subsequent steps. Xylene was used as a clearing agent. The organs were embedded in paraffin, sections were cut into 2–5μm thickness using rotary microtome and were stained with hematoxylin and eosin. Pictures of the section were taken with OMAX 40X-2500X LED Digital Trinocular Microscope with USB digital Camera (Irvine, California, USA) and analyzed with ImageJ 153a software.

### Extraction and clean up for Gaz Chromatography–Mass Spectrometry (GC-MS)

Ten grams of the mix soft part of four snails coming from each collected group were mixed with 10 ml of acetonitrile, 2 g of MgSO4 and 0.5 g of NaCl. The mixture was vortexed for 1 min, and shaken for 15 min. Then, the samples were centrifuged at 3000 rpm for 5 min. One ml of the supernatant was mixed with 100 mg of Primary Secondary Amine (PSA) and 400 mg of MgSO4, vortexed for 2 min, and centrifuged as described above. The solvent was filtered on 0.25 μm thickness filter disk and subjected for GC-MS determination [43].

### Gaz Chromatography–Mass Spectrometry (GC-MS) determination

Agilent Technologies 7890 GC system-coupled with MS-5977A MSD, Japan was used at the Faculty of Agriculture, Damanhour University, Egypt. GC-MS instrument with electron impact (EI) ionization, autosampler (AS), and computerized instrument control/data collection was used. Injection volume (2 μl) as spitless mode at 250 °C was conducted. An analytical column (30 m, 0.25 mm id, and 0.25 μm thickness of 5% phenyl methyl polysiloxane) was used and Helium was used as a carrier gas at a rate 1ml/min. Temperature program was started at 100 °C and ramped to 280 °C at a rate 10 °C/min. Software program used to estimate the output data.

### Analytical method validation

All laboratory glassware were soaked for 12 hours in acid solution, washed carefully, purged with distilled water and acetone before use. All used chemicals were analytical grad. The limits of detection (LODs) ranged from 0.005 to 1.0 ng/ml for the examined pesticides. Extraction and clean up procedures were evaluated through recovery experiment which ranged from 94.0 to 95.5%. Also, procedural blank with no pesticides was performed. The calibration standards were done with coefficient of regression (R) ≥0.995. To employ reproducibility, each sample was duplicated.

### Statistics

Mean of the weight and size of the animals were compared together, as well as Comparison of Vacuoles quantity estimation within the Eosin-hematoxylin-stained kidney by using Welsh’s One-way Analysis of variance with the p value p≤0.05 (ANOVA) with GraphPad Prism 9 software.

### Ethics statement

All experiments therein have been conducted in accordance with current scientific bioethics law of Cameroon. All organisms unexposed to toxicants (i.e. unused and untested were given as food). Njombe and Ebodje sampling site locations are owned by the Institute of Agricultural Research for Development. The sampling site in Kribi rural is owned by the company SOCAPALM and had approved to perform the sampling. Concerning the remaining sampling location, Dibombari is not privately-owned or protected in any way and snails sampling at these locations did not require any specific permit. The field studies did not involve endangered or protected species.

## Results

### Result of collection

Two types of snail species were collected from the four sampling sites: *Archachatina marginata* and *Achatina fulica*. *Archachatina marginata* was the main species found at all the sites (Table 1). Further analyses were done on *Archachatina marginata*.

**Table 1:** Table of snails collected.

Animal Strain		*Archachatina marginata*	*Archachatina fulica*
Collection site			
**Weakly agricultural site**	**Dibombari**	32	0
	**Ebodje**	18	2
**Strongly Agricultural site**	**Njombe**	38	5
	**Kribi rural**	36	1

Collection was performed in four sites (weakly vs strongly pesticides exposed). Two types of snails were found: *Archachatina marginata* and *Achatina fulica*.

### Pesticide measurements on snail soft part

**Fig 2:**
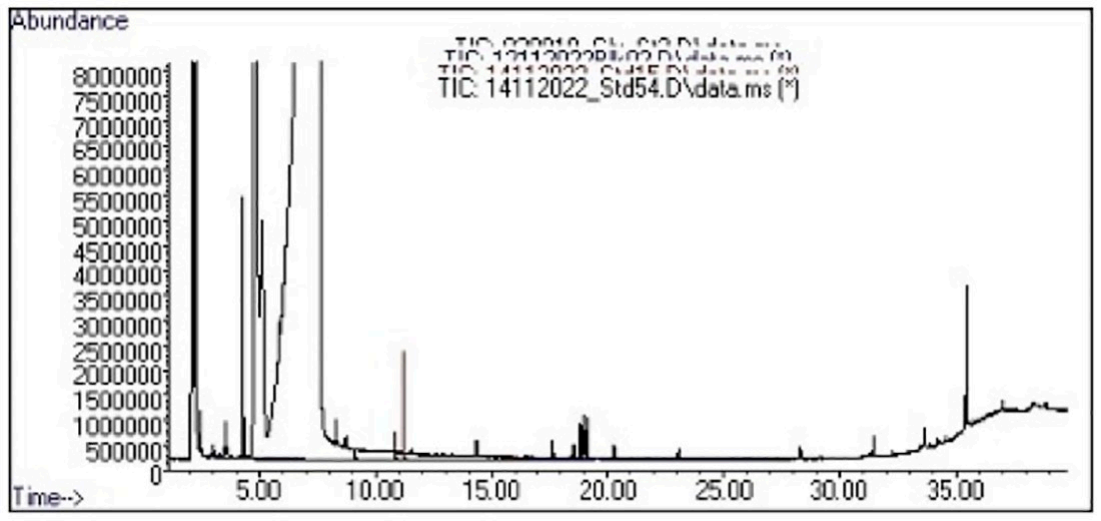
Chromatogram of the pesticides measured and analyzed in *Archachatina marginata* by GC-MS analysis. 10 g of the homogenate of four snails soft part coming from each collected group were used for mass spectrometry analysis. Peaks are the recorded mass spectra of each pesticide with corresponding retention time analyzed by the software GC-MS instrument used. The presence of several peaks on this chromatogram is due to the various degradation products and/or isomer of each pesticide molecule.

Pesticides were measured on the soft part of snails in order to evaluate if they can be transferred to the flesh of the snail. The analysis of the chromatogram (Fig 2) showed in Table 2 that glyphosate is present in animals from all collection sites. In contrast, metalaxyl was not detected in animals from any of the collection sites. For cypermethrin, it was found in Njombe, Ebodje and Kribi rural samples but absent from Dibombari (Table 2).

**Table 2:** Table of Pesticides residues levels (μg/g tissue) in whole body homogenate of collected *Archachatina marginata* snails.

Pesticides	Glyphosate	Metalaxyl	Cypermethrin
Location			
**Dibombari**	1.01399±0.7	ND	ND
**Ebodje**	1.14834±0.812	ND	2.516±1.677
**Njombe**	0.35497±0.251	ND	3.035±2.037
**Kribi Rural**	0.09899±0.07	ND	10.90217±7.709

10 g of the homogenate of four snails soft part coming from each collected group were used for mass spectrometry analysis. Value showed in the table are the mean of measured values coupled with their Standard Error of the Mean (SEM) obtained subsequently the analysis of pesticides peaks by the software of the GC-MS instrument. ND: not detected.

### Effect of pesticide exposure on the general physiology of animals

**Fig 3:**
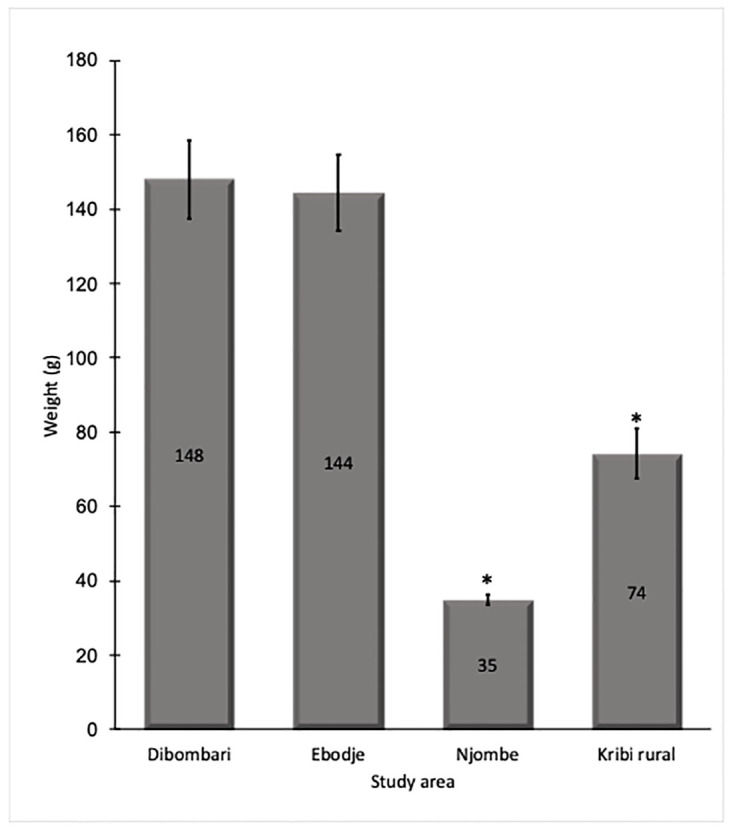
Pesticide exposure interfere with the weight of snails *Archachatina marginata*. Weight of collected *Archachatina marginata* snails were measured, compared together using Welsh’s One-way ANOVA and presented as histogram. Star indicates a statistically significant difference (* p≤0.05).

**Fig 4:**
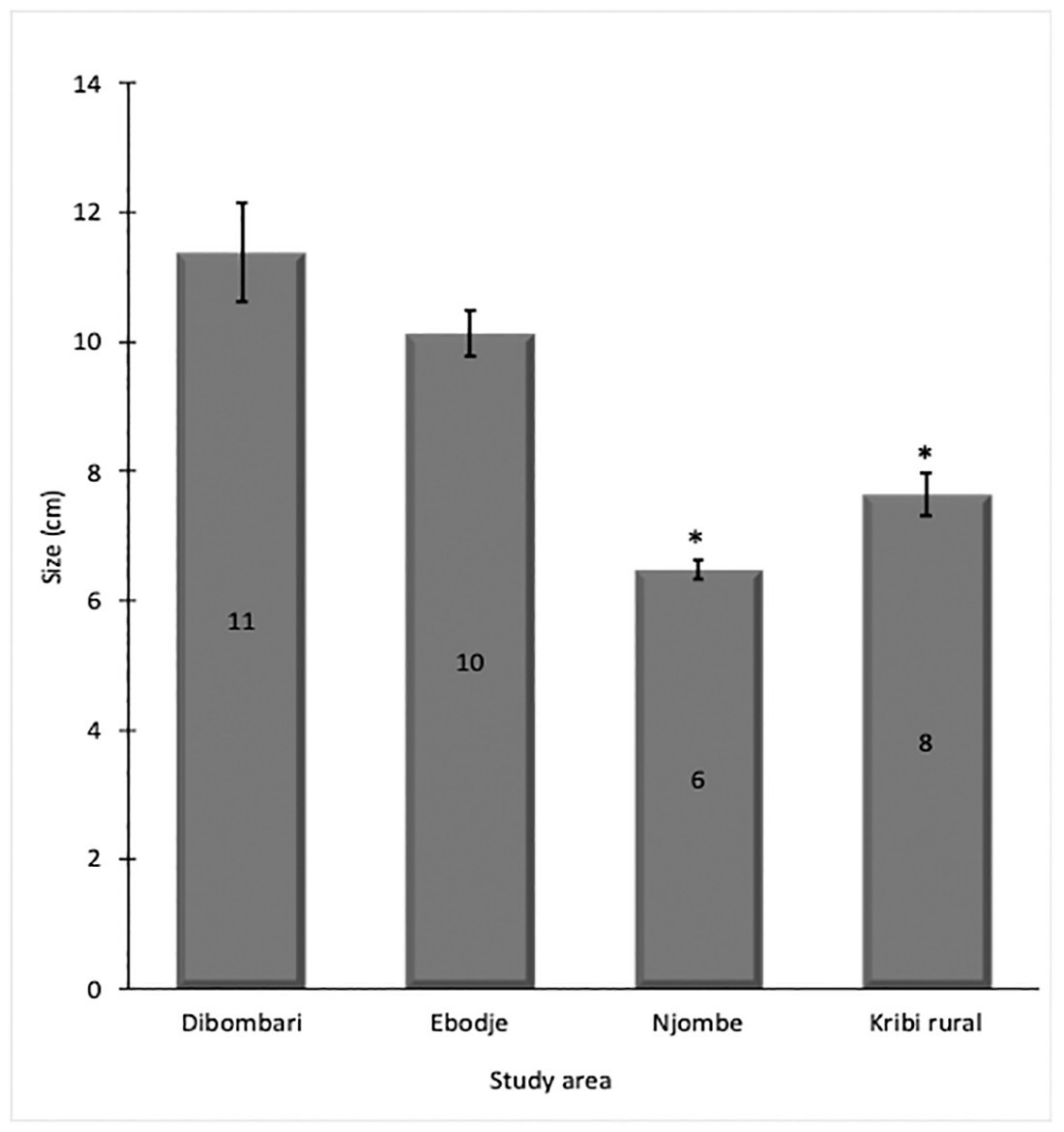
Pesticide exposure interfere with the size of snails *Archachatina marginata*. Size of collected *Archachatina marginata* snails were measured, compared together using Welsh’s One-way ANOVA and presented as histogram. Star indicates a statistically significant difference (* p≤0.05).

**Fig 5:**
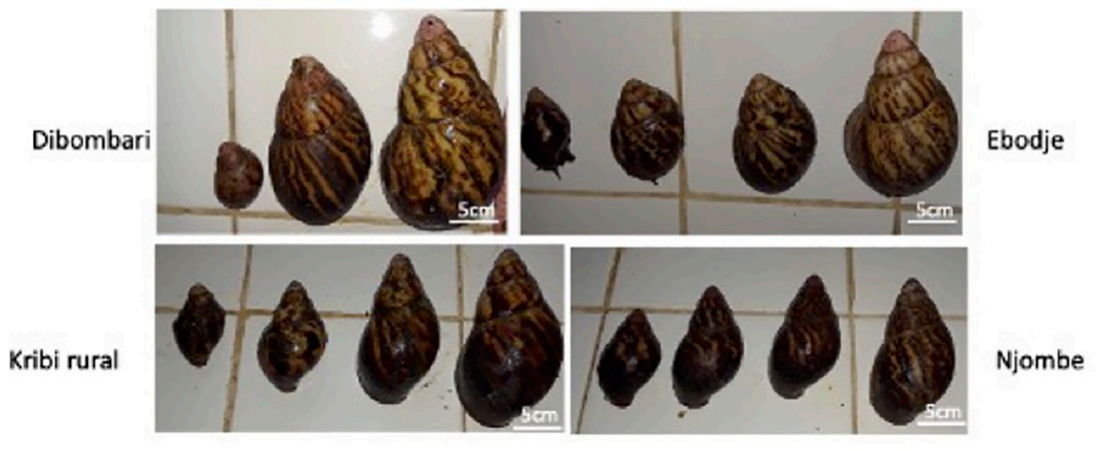
Snails coming from Dibombari are bigger than those from other sites. Picture of the different sizes of *Archachatina marginata* snails collected: Diverse sizes of *Archachatina marginata* snails (from the bigger to the smaller snail) coming from each site was put beside each other to have a visual comparison of histograms results.

Foot and shell, weight and length were examined to assess if the exposure to the different pesticides could affect the general physiology of *Archachatina marginata* snails. Results showed that snails collected in the less-exposed areas are heavier than those coming from more pesticide exposed areas: Dibombari 148 g and Ebodje 144 g versus Kribi rural 74 g and Njombe 35 g (Fig 3). It was also observed that the less exposed snails are taller than those more exposed: Dibombari 11 cm and Ebodje 10 cm versus Njombe 6 cm and Kribi rural 8 cm (Figs 4 and 5).

### Effect of pesticide exposure on ovo-testis structure of snails

**Fig 6:**
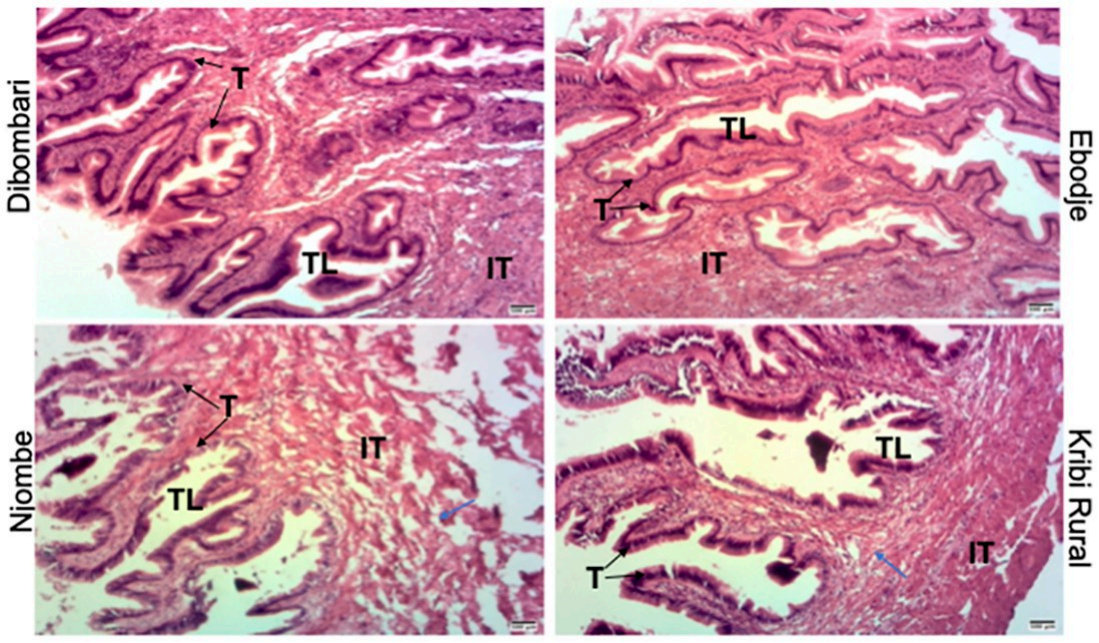
Pesticides exposure disrupts *Archachatina marginata* ovo-testis. Microphotograph of the Ovo-testis. Ovo-testis was excised out from the animals, sectioned and stained with eosin-hematoxylin. The structures of the conjunctive tissues were compared together. Njombe has the conjunctive tissue disrupted than the others. Blue arrow is showing the conjunctive tissue disruption. IT: Interstitial tissues, T: Tubules, TL: Tube Lumen.

Histological sections of the hermaphrodite reproductive gonad (ovo-testis) of *Archachatina marginata* snail was analyzed to determine whether pesticide exposure could disrupt their structures. Result of eosin-hematoxylin-stained sections showed a distinct disruption of the interstitial tissue on snail coming from Kribi rural and Njombe areas. The disruption is more noticeable with sections of snails collected from Njombe (Fig 6).

### Pesticide exposure on the structure of the kidney of snails

**Fig 7:**
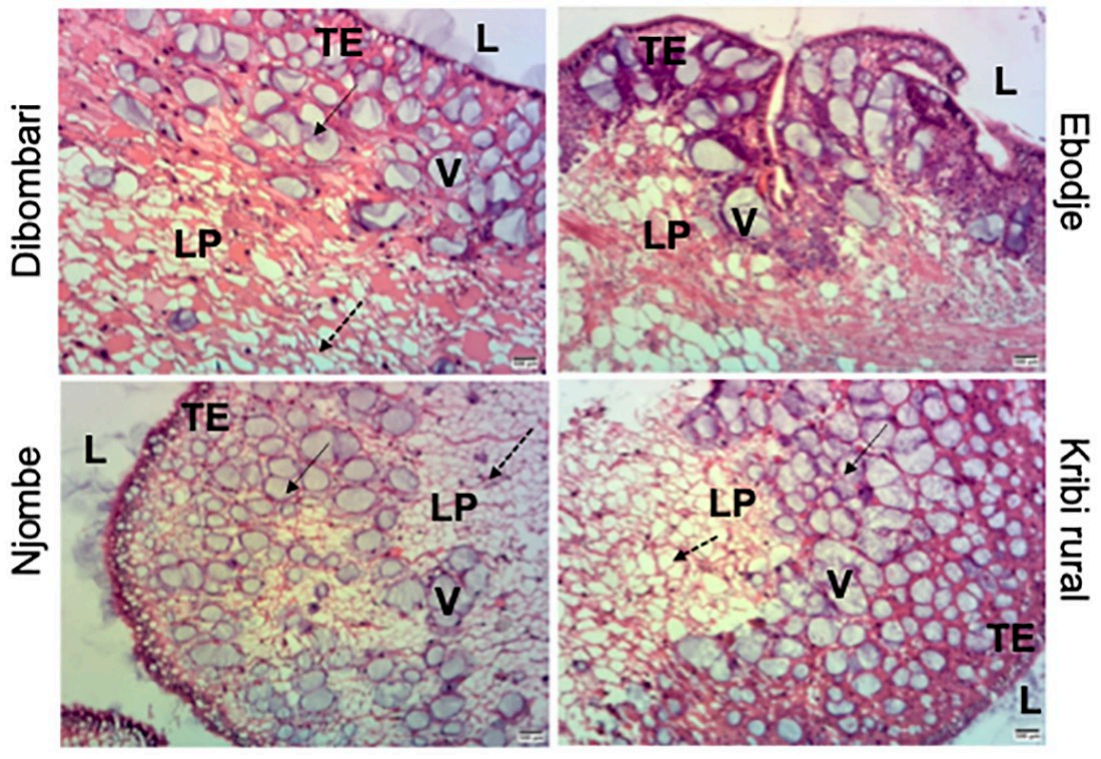
Pesticides exposure disrupts *Archachatina marginata* kidney. Microphotograph of the kidney. Kidney was excised out from the animals, sectioned and stained with eosin-hematoxylin. The structures of the lamina propria tissue and the presence of vacuoles were compared together. Njombe and Kribi rural have the conjunctive tissue disrupted than the others. As well the presence of a lot of vacuoles is visible in sections of the above-mentioned sites. The plain Arrow is showing the area with multiple vacuoles and the dash arrow is showing where Lamina propria is more disrupted. V: Vacuoles, LP: Lamina propria, TE: transitional epithelium.

**Fig 8:**
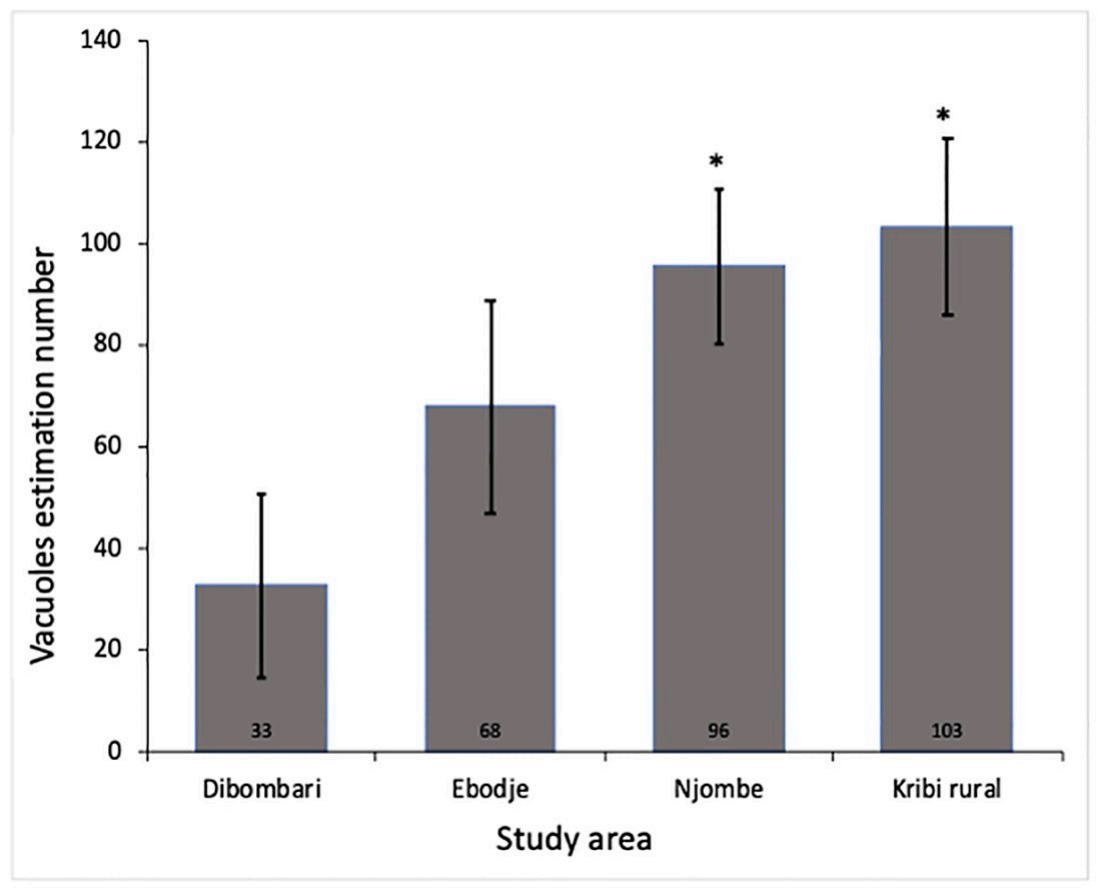
Pesticides exposure increase vacuole quantity within kidney. Kidney was excised out from the animals, sectioned and stained with eosin-hematoxylin. Quantity of vacuole were counted, then compared together using Welsh’s One-way ANOVA and presented as histogram. Star indicates a statistically significant difference (* p≤0.05).

Histological sections of the kidney of *Archachatina marginata* snails were analyzed to determine whether pesticide exposure can impair their structures. Results of eosin- hematoxylin-stained sections of kidney showed a noticeable disruption of the structure of kidney lamina propria in samples from Njombe and Kribi rural areas (Fig 7). When quantity of vacuoles was compared together, a statistically significative increase in vacuoles were observed between samples coming from Njombe and Kribi rural areas when compared to Dibombari samples but not with Ebodje (Fig 8).

## Discussion

The aim of the present study was to evaluate pesticide contamination and its impact on *Achatinadea* snails.

Animal collection showed that the snail species *Archachatina marginata* and *Achatina fulica* are the two species found in the collection areas. This result is supported by the survey of Tsayo *et al* that made a census of the types of snails found in the department of Mfoundi, Cameroon Center Region which is a region whose climatic conditions are similar to the study area [13]. On the other hand, in their study a similar number of individuals from the different species of snails was found whereas in our present study we have collected more *Archachatina marginata* snails. There are studies that mentioned *Achatina fulica* as invasive species [44–46]. We therefore expected to have individuals from this species in our collection, especially since they are the least consumed species in Cameroon, but we harvested less comparing to *Archachatina marginata*.

One of our hypotheses was that Cameroon most used pesticides are transferred into the flesh of snails. Previous studies with land snails including *Achatinadea* snails revealed the presence of heavy metals [20, 25, 37, 39] and pesticides [21, 24, 36, 47–49] on their flesh. Our present study also found that exposure to pesticides leads to their transfer into the flesh of *Archachatina marginata* snails. Indeed, the mass spectrometry results showed that glyphosate is present in the flesh of all the animals collected, regardless of the degree of agricultural activity. Indeed, glyphosate is an herbicide commonly used in most industrial or family-type fields to eliminate weeds in Cameroon. In contrast, metalaxyl was not detected in the sample of animals from any of the collection sites. Can probably be explained by the fact that metalaxyl is a fungicide used to fight against brown rot in cocoa farming and the fields where we collected snails are oil palm (Kribi rural) and plantain (Njombe) fields, not cocoa farms. Animals in Kribi rural and Njombe are not likely to be exposed to the metalaxyl. Moreover, since the recent prohibition of metalaxyl fungicide in Cameroon, several farmers tend more and more to abandon its use. Regarding cypermethrin, it was found in Njombe, Ebodje and Kribi rural samples but not in samples from Dibombari (Table 2). Besides, this study was focused on the presence/absence of pesticides in the *Archachatina marginata* snail which could be an indication of the transfer of these pesticides in the flesh of the animals collected. More work is needed to determine if consumption of contaminated snails could be a hazard for human health.

We also evaluated whether there is any impact of pesticides on *Archachatina marginata* snail physiology. We found that snails from pesticide heavily exposed areas are smaller in size and weight. This result suggests that the low weight and size could be a consequence of pesticide exposure. Indeed, the study of Wandan *et al* showed that exposure to high doses of the pesticide endosulfan do slightly negatively affect the weight of *Achatina achatina* snails which also belong to the *Achatinadea* group [50]. Other studies on different land snails have shown a correlation between snail growth and exposure to certain heavy metals like copper and lead [49, 51, 52]. Another hypothesis that can explain the low weight and size of snails from the high pesticide areas is that these areas also have more human activity, and the collection of snails for consumption is also higher. The small size of the snails could simply be due to the fact that they do not have enough time to grow and reach sizes seen in the less exposed areas of Ebodje and Dibombari that are wild forest areas. Assessing the impact of these pesticides on animal growth and the levels of growth hormone, in controlled laboratory conditions is warranted.

Finally, histological analysis of two organs, the kidney and the ovo-testis, showed a significant disruption in their structures. The disruption is more pronounced on the sections of the animals collected at Njombe which is heavily exposed to pesticides. Our results are in agreement with several studies carried out on animals exposed to heavy metals [38, 39] and certain pesticides such as glyphosate and chlorate pesticide [47, 53]. It would be interesting to evaluate the impact of these pesticides on the function of these two organs by measuring reproductive hormones and kidney enzymes.

## Conclusion

The increase in the use of pesticides is a real problem in Africa, especially since their use is not well regulated. In order to find solutions to limit this pollution, our study was able to highlight that *Archachatina marginata* snails can be biological indicators to monitor soil pesticide pollution. Indeed, we confirmed the transfer of some pesticides in the flesh of snails caught in the usual collection areas and used to supply the market vendor. In addition, the results of this study showed that since there are pesticides in the meat of these animals, it represents a potential means of human contamination throughout the consumption of contaminated meat. The originality of our study lies in the use of a model that is very popular in Cameroon in real condition meaning: direct collection in usual areas by focusing on pesticides less *vs* highly contaminated sites. In contrary to other studies on *Archachatina marginata* that were performed in controlled areas. Furthermore, our result indirectly supports *Archachatina marginata* breeding in controlled area. Which is favorable for the specie conservation in the wild.

## Supporting information

S1 File Row data of morphology analysis. Weight and size of *Archachatina marginata* snail were taken and then statistically analyzed using Welsh’s One-way Analysis of variance with the p value p≤0.05 (ANOVA).
(XLSX)

S2 File Row data of GPS values measured at the collection sites. Coordinates of the four collection areas were taken by a GPS at night between July to September 2020 using an adaptation of a square kilometer method.
(XLSX)

S3 File Row data of mass spectrometry analysis. Value showed in the table are the mean of measured values coupled with their Standard Error of the Mean (SEM) obtained subsequently the analysis of pesticides peaks coming from the homogenate of collected snails’ soft part by the software of the GC-MS instrument.
(XLSX)

S4 File Row data of histological analysis. Microphotographs of the Ovo-testis and Kidney that were excised out from the collected *Archachatina marginata* snails, sectioned and stained with eosin-hematoxylin.
(PDF)
