# Supplementary material for: Impact of pesticides exposure on Archachatina marginata snails in four Cameroon monomodal rainforest sites
Source: PLoS One. 2024 Mar 4;19(3):e0297369. doi: 10.1371/journal.pone.0297369 (PMC10911591; doi:10.1371/journal.pone.0297369)
Supplement: S4 File — Microphotographs of the Ovo-testis and Kidney that were excised out from the collected Archachatina marginata snails, sectioned and stained with eosin-hematoxylin. (PDF) [file pone.0297369.s004.pdf]

Kidney

Dibombari

Snail 1

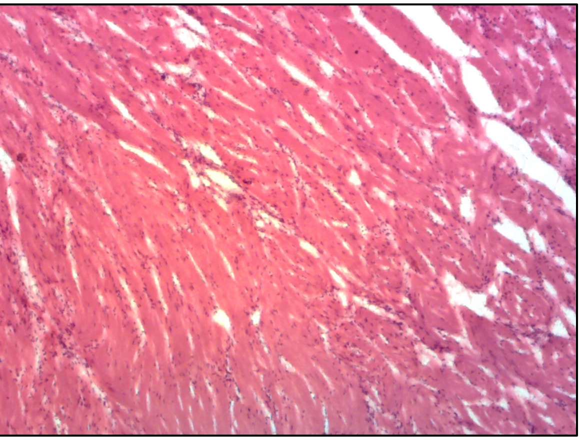

View 1

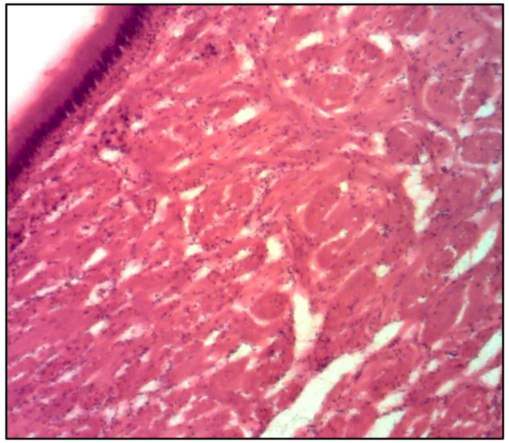

View 2

Snail 2

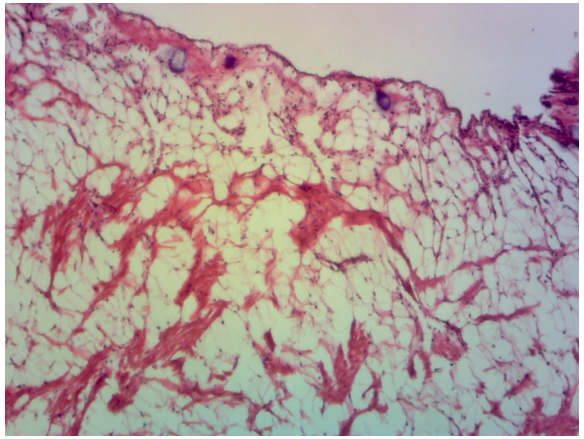

View 1

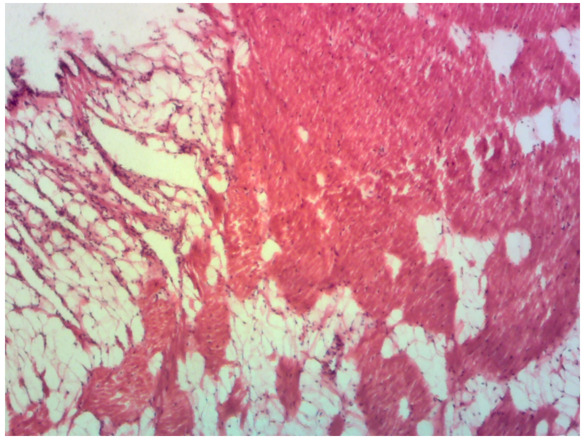

View 2

Snail 3

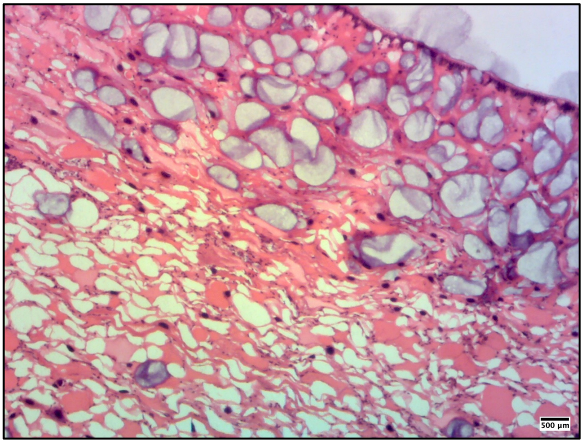

View 1

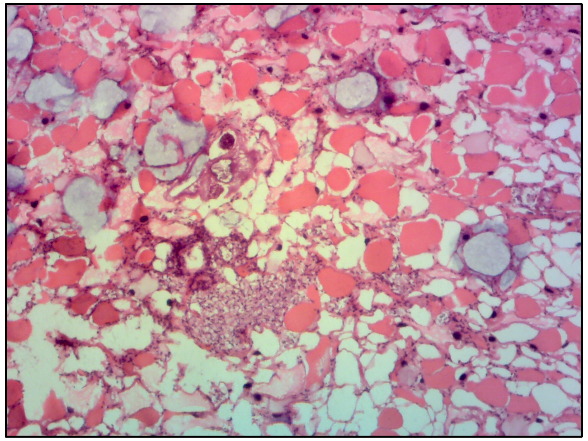

View 2

Dibombari

Ovotestis

Snail 1

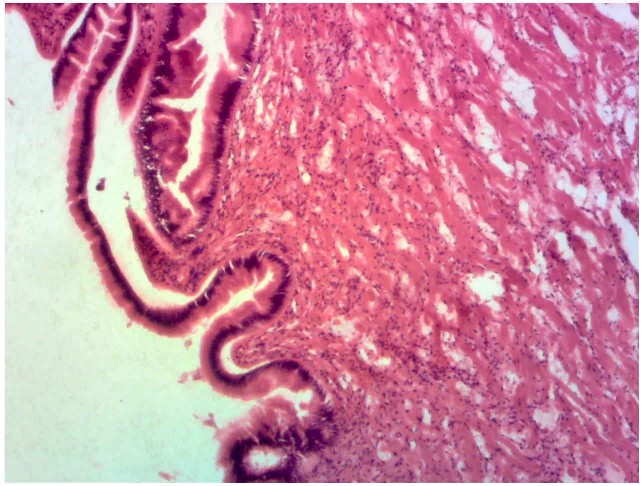

View 1

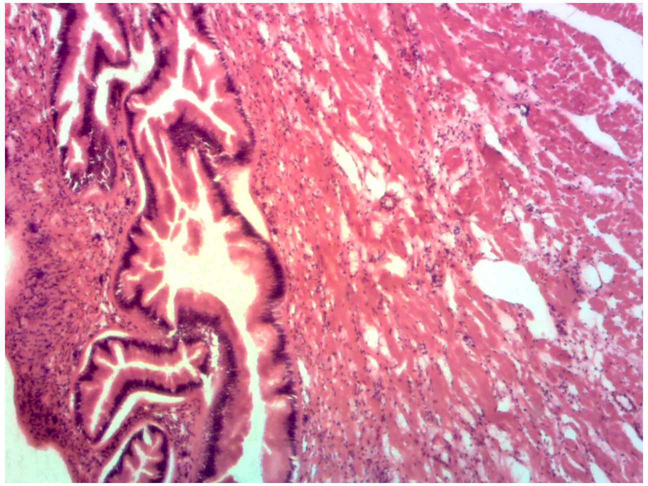

View 2

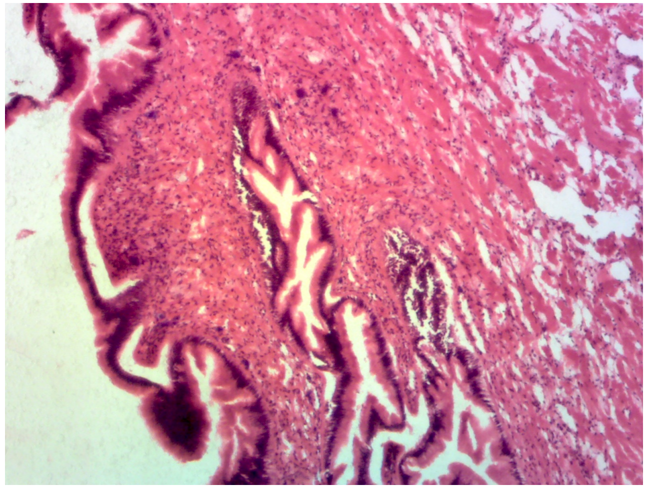

View 3

Snail 3

View 1

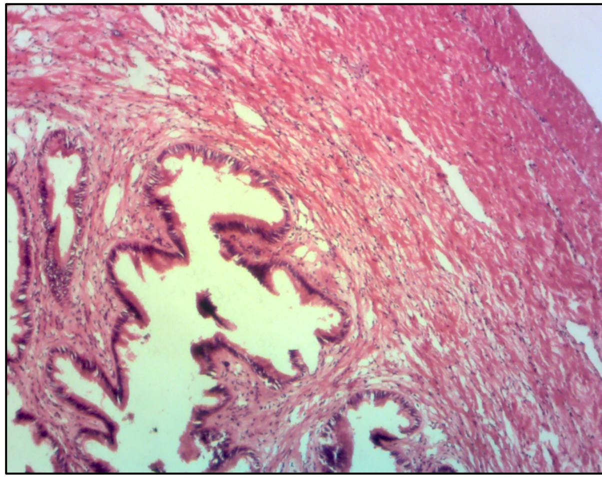

Snail 2

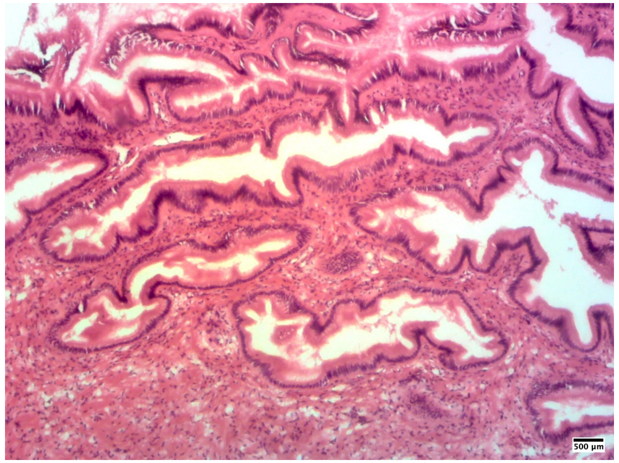

View 1

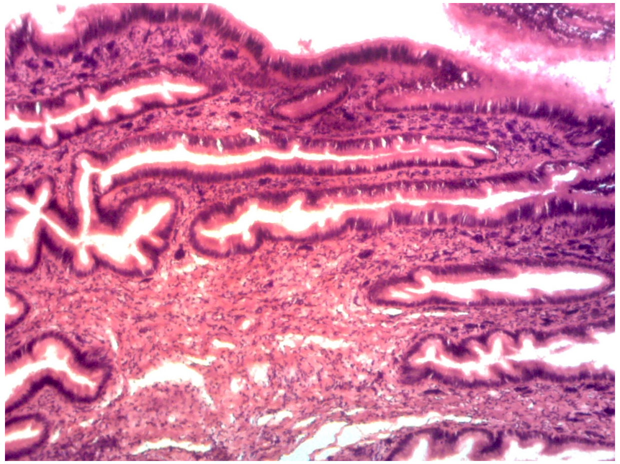

View 2

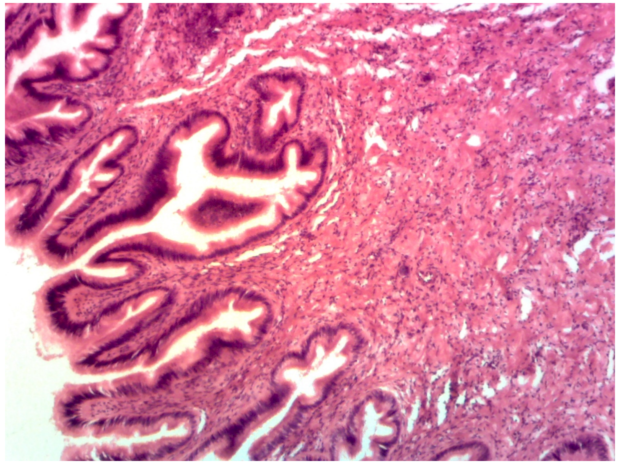

View 3

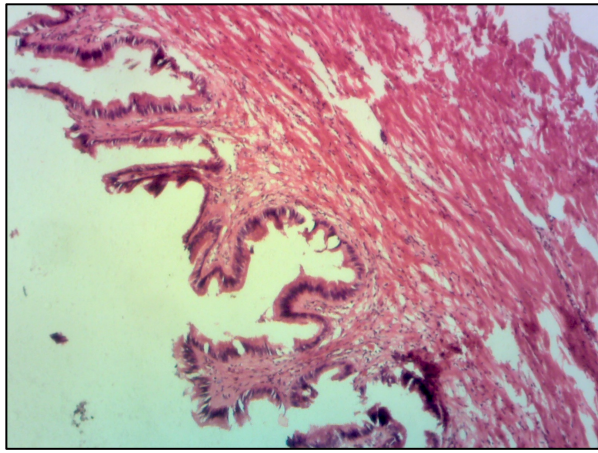

View 2

Kidney

Snail 1

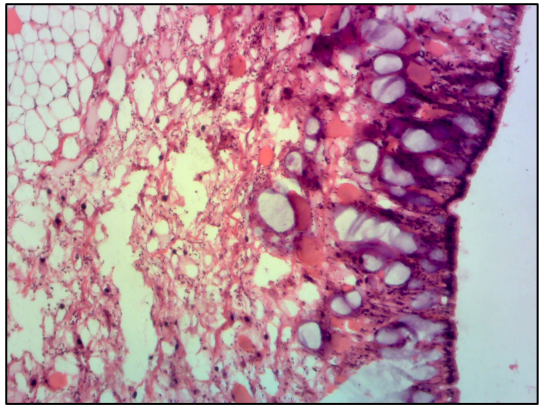

View 1

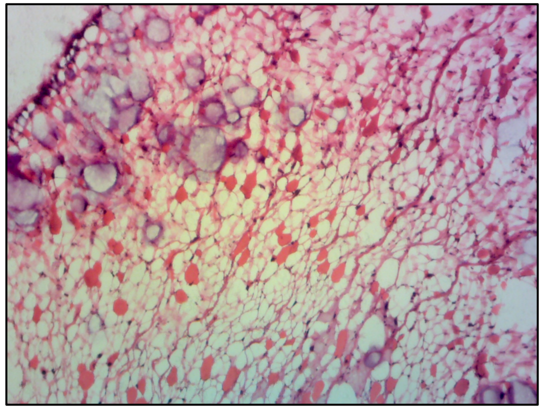

View 2

Ebodje

Snail 2

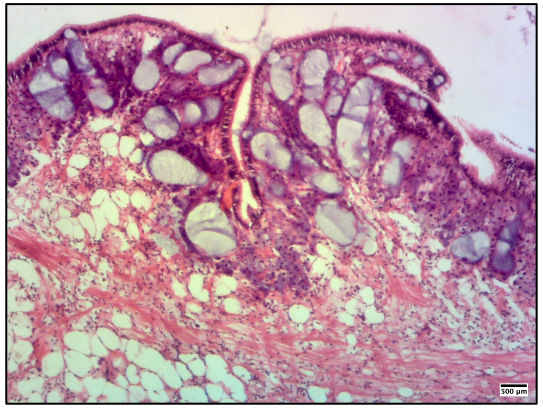

View 1

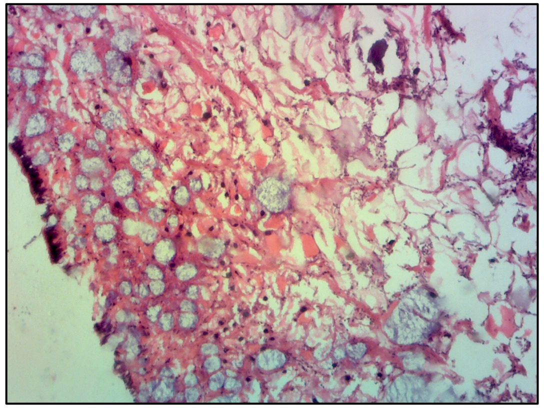

View 2

Snail 3

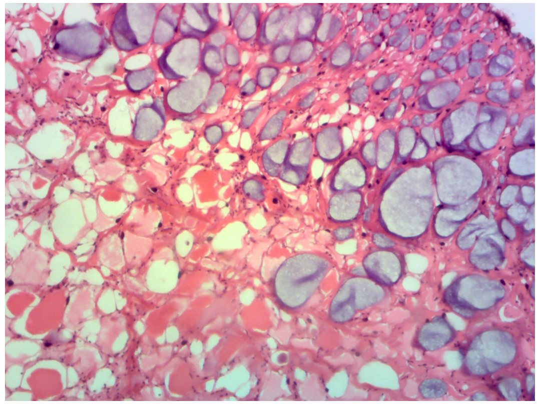

View 1

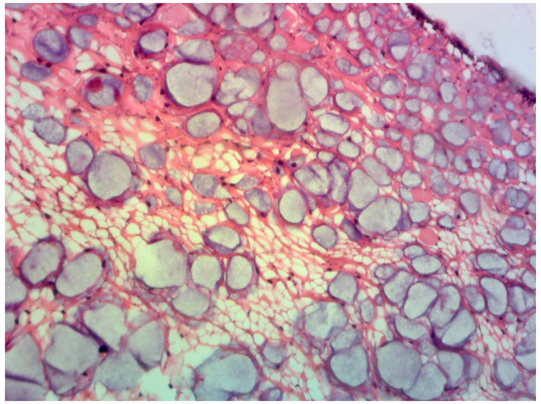

View 2

Ovo-testis

Ebodje

Snail 1

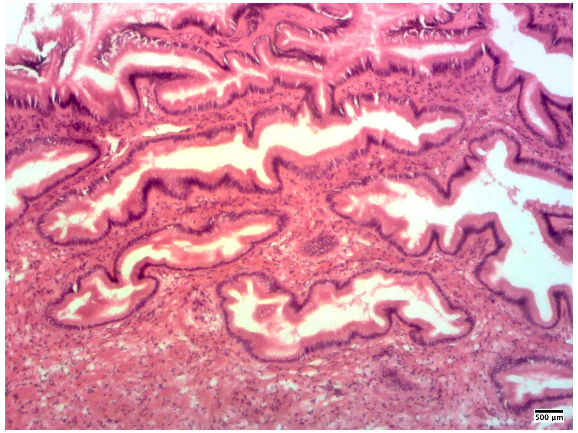

View 1

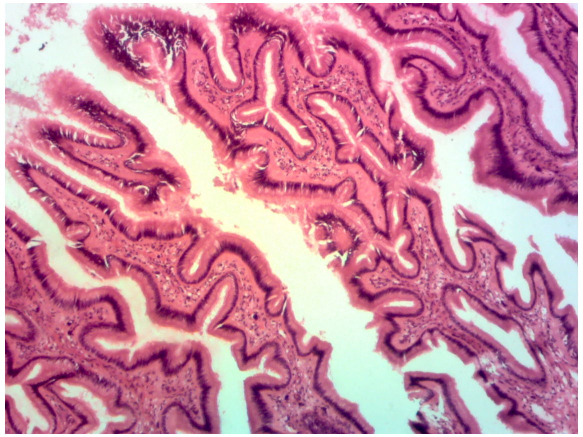

View 2

Snail 2

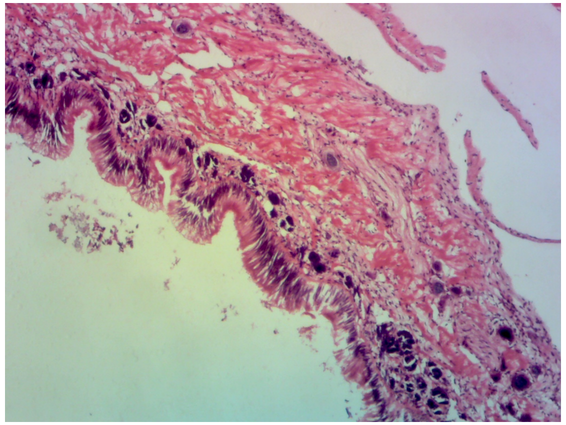

View 1

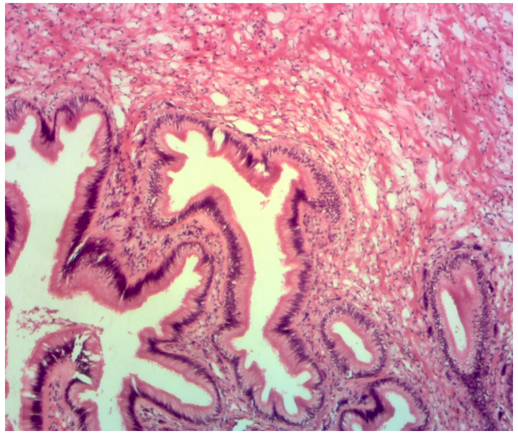

View 2

Snail 3

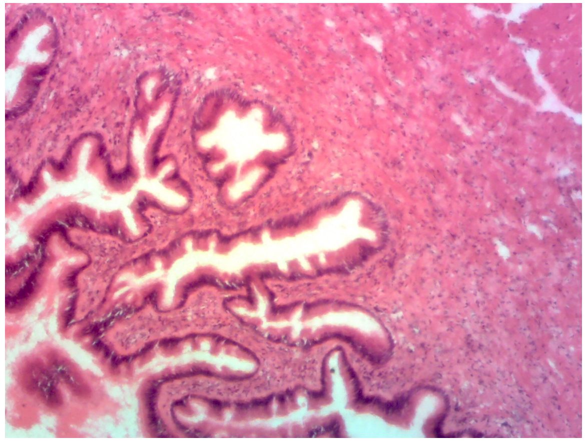

View 1

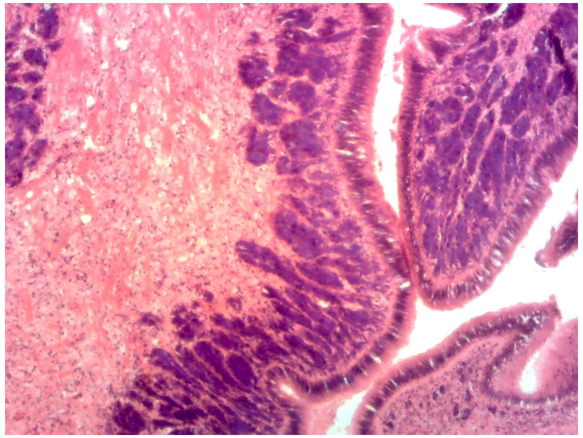

View 2

Kidney

Kribi rural

Snail 1

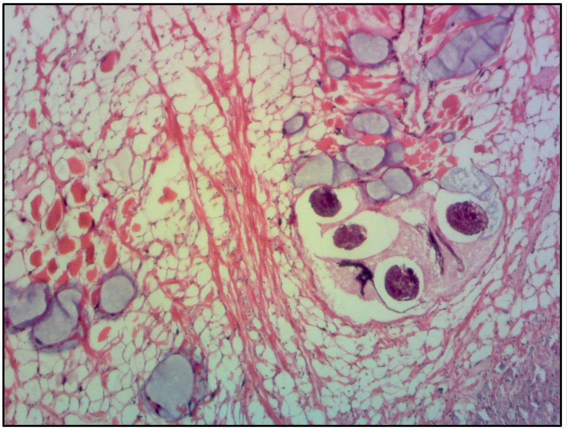

View 1

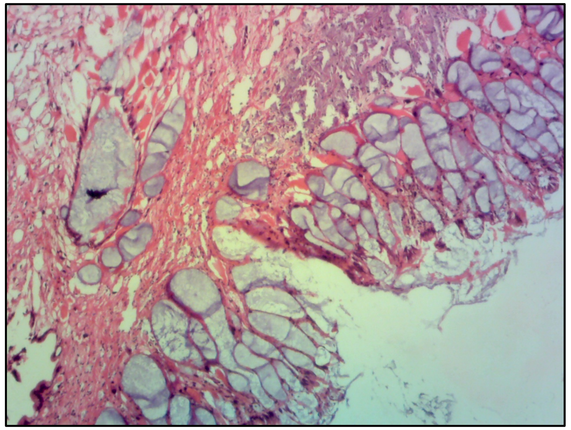

View 2

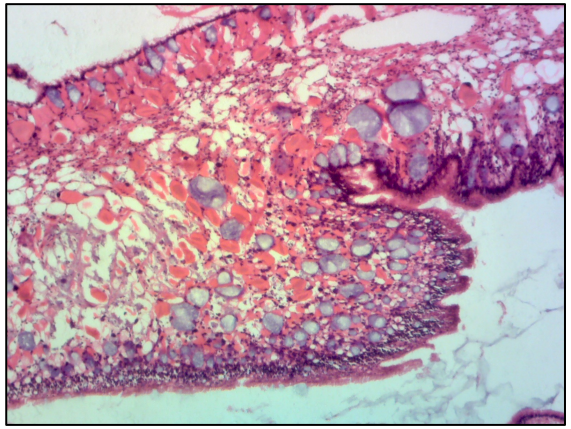

View 3

Snail 2

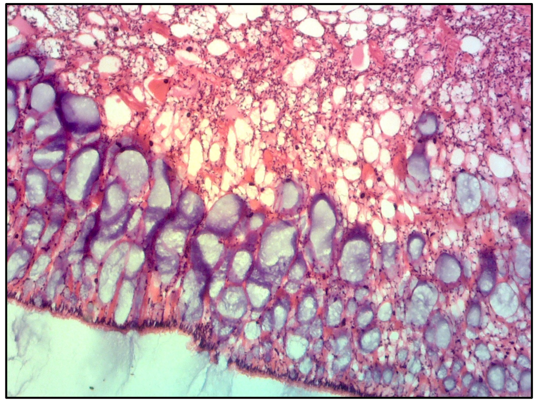

View 1

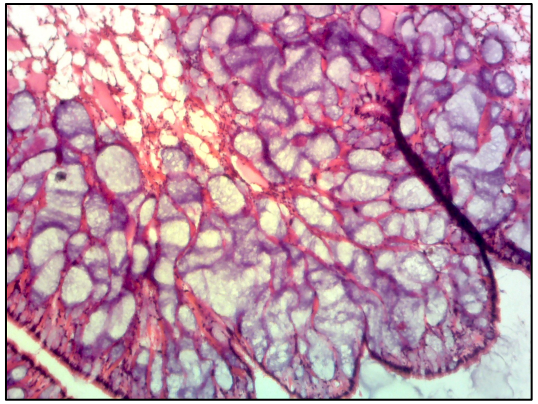

View 2

Snail 3

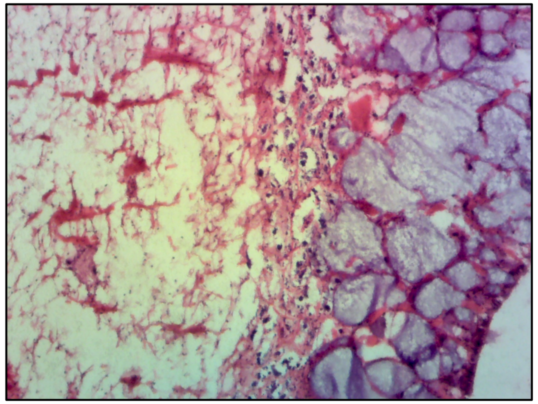

View 1

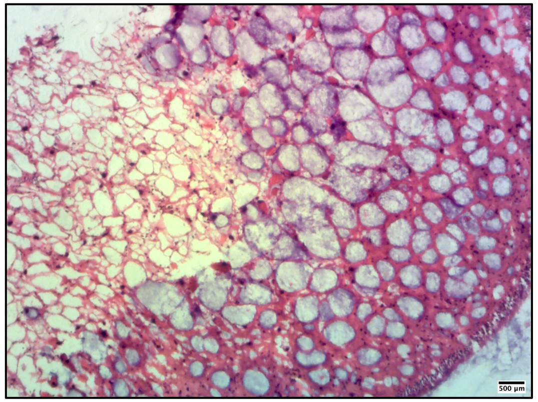

View 2

Kribi rural

Snail 1 Ovotestis

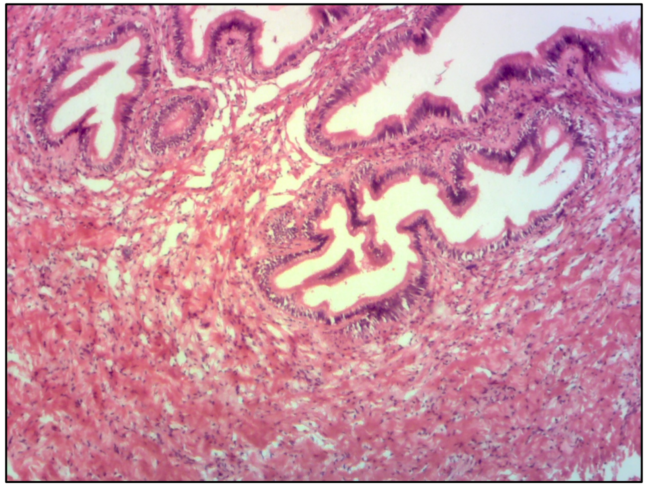

View 1

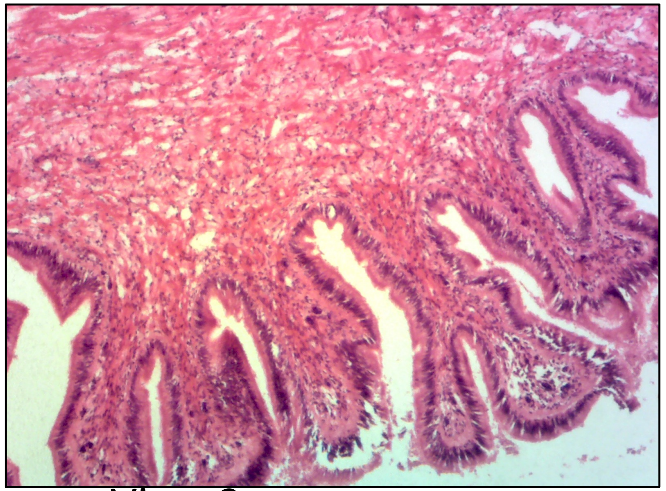

View 2

Snail 2

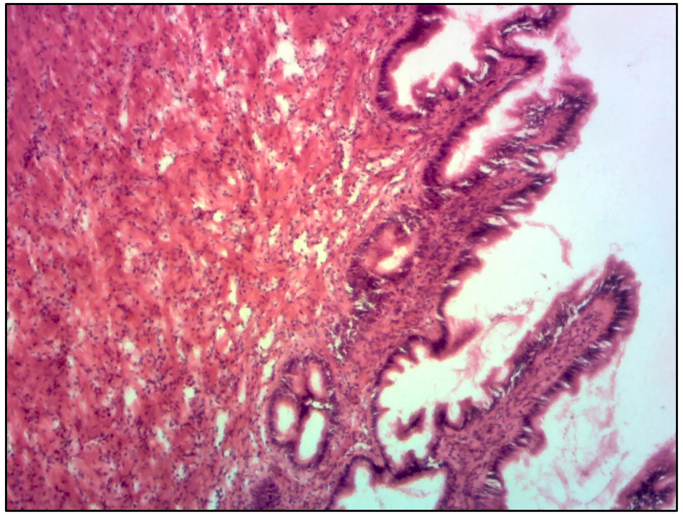

View 1

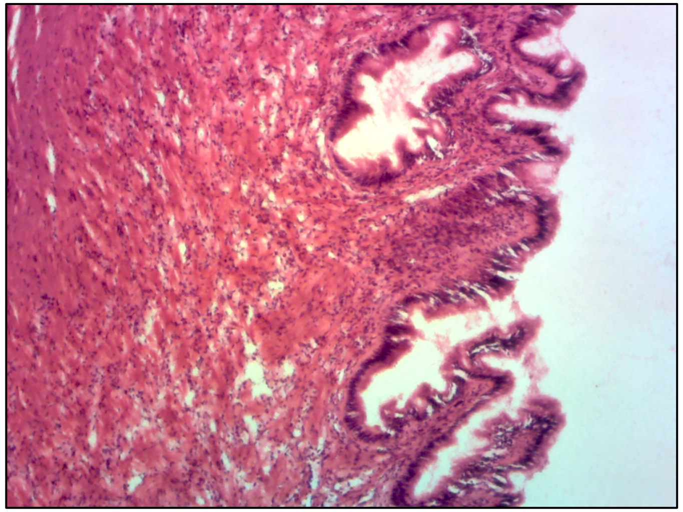

View 2

Snail 3

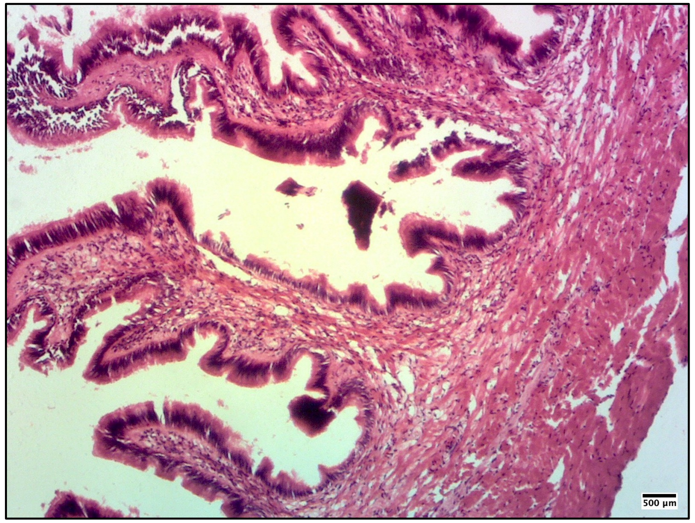

View 1

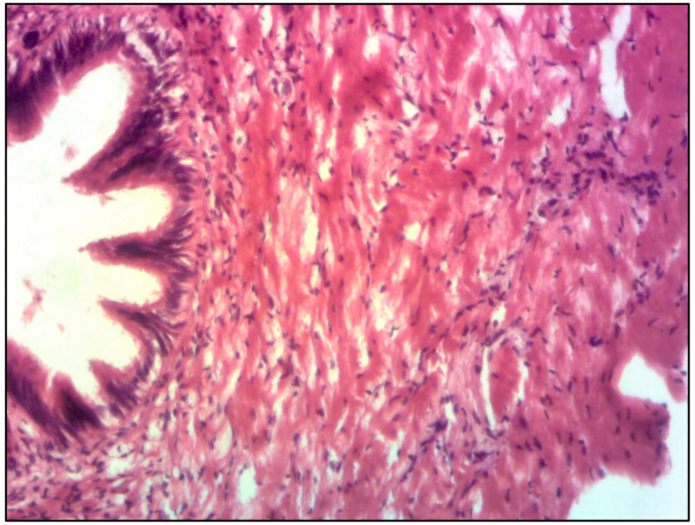

View 2

Kidney

Njombe

Snail 1

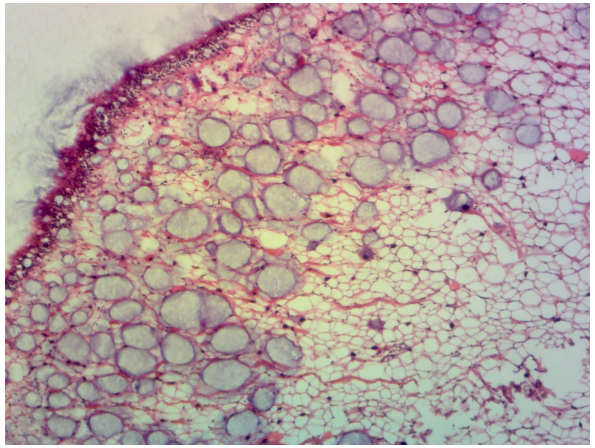

View 1

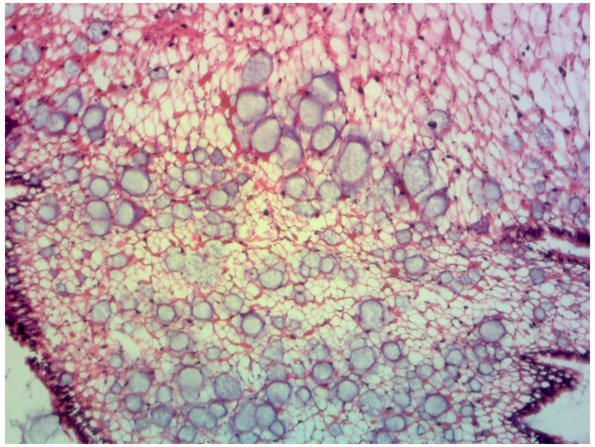

View 2

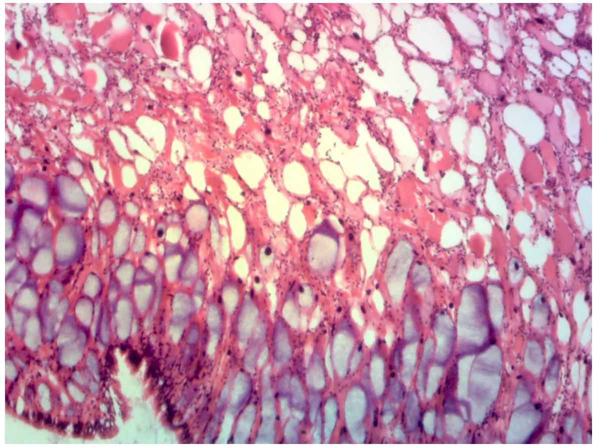

View 3

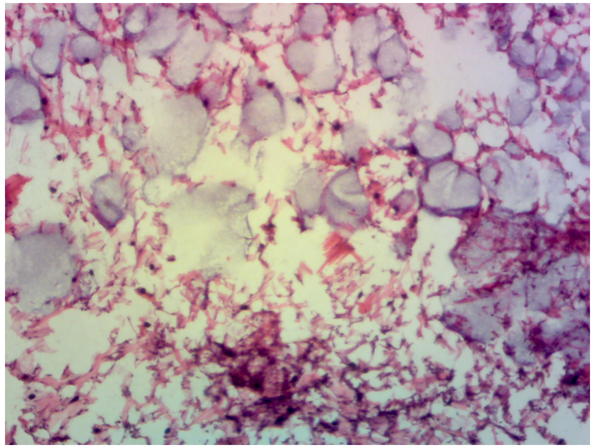

View 1

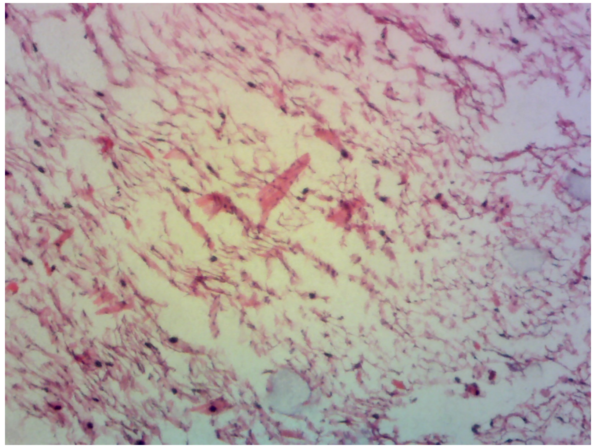

View 2

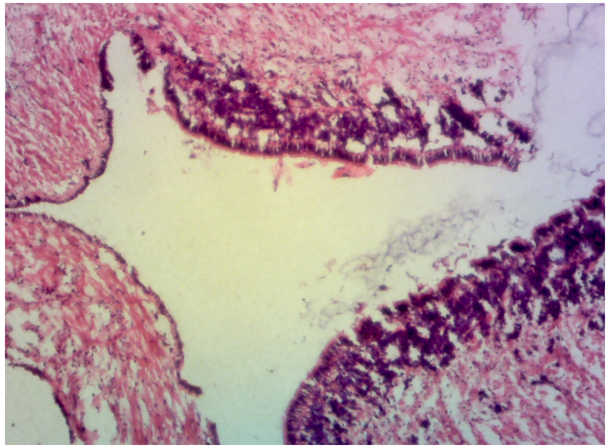

View 3

Snail 3

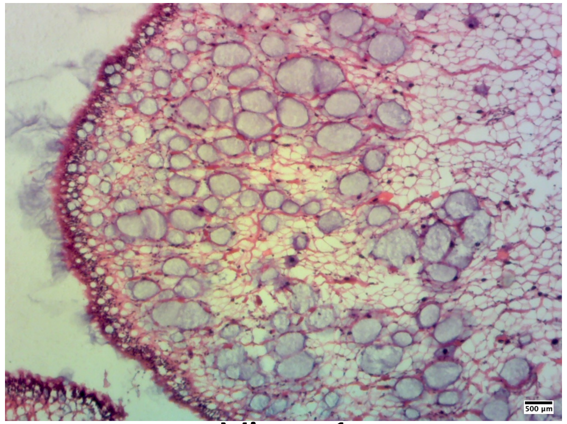

View 1

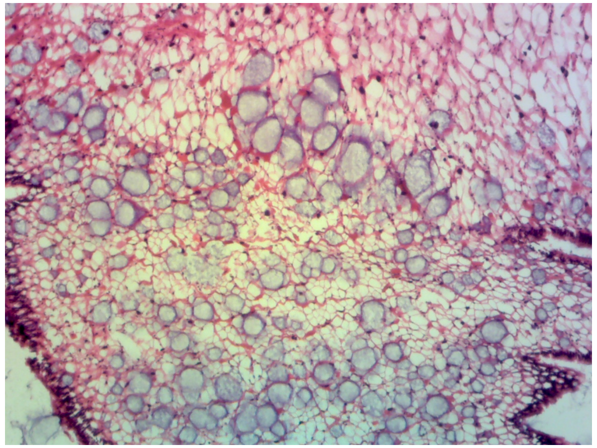

View 2

View 3

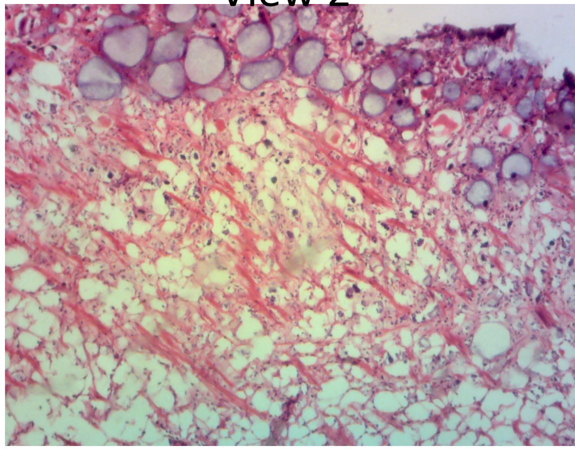

Njombe

Ovotestis

Snail 1

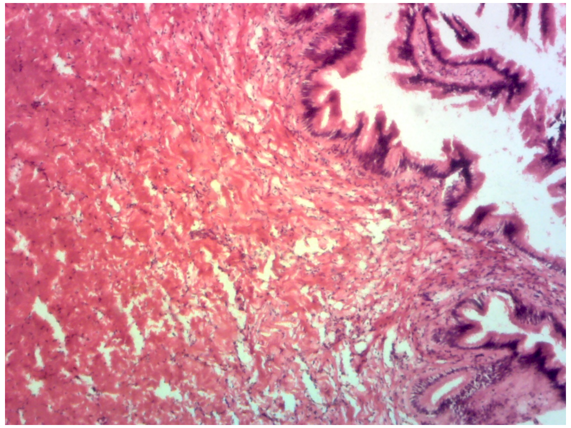

View 1

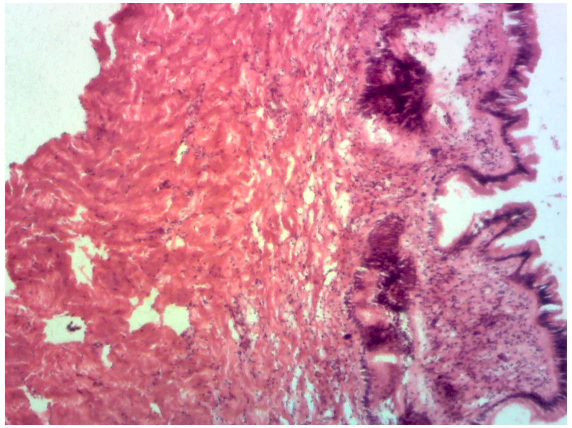

View 2

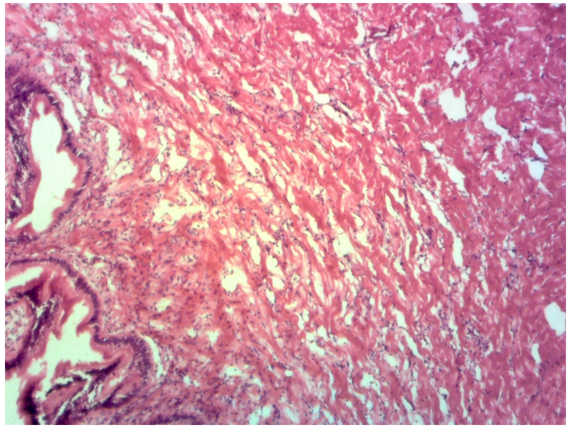

View 3

Snail 3

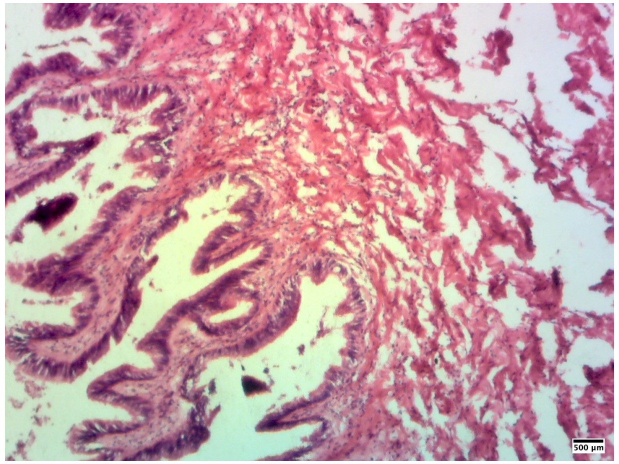

View 1

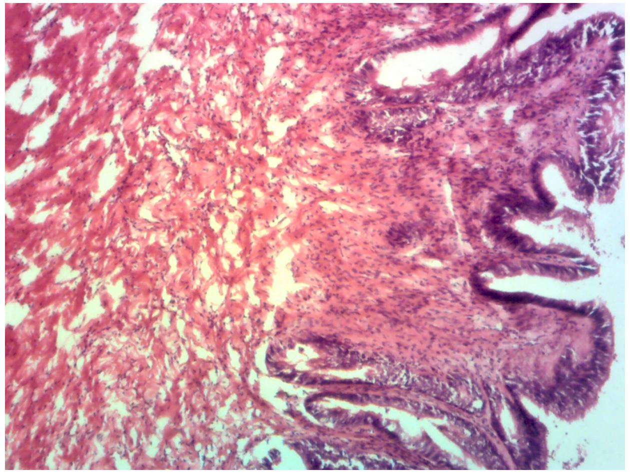

View 2

Snail 2

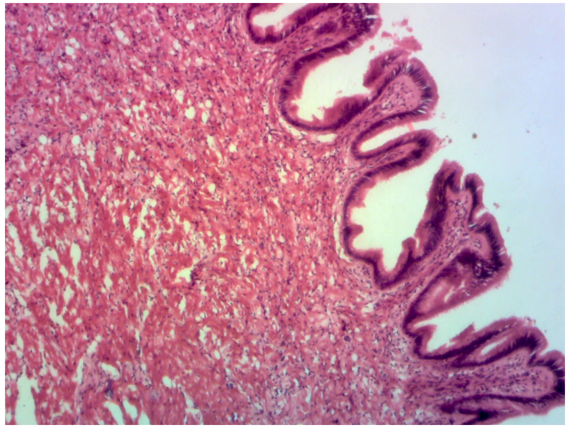

View 1

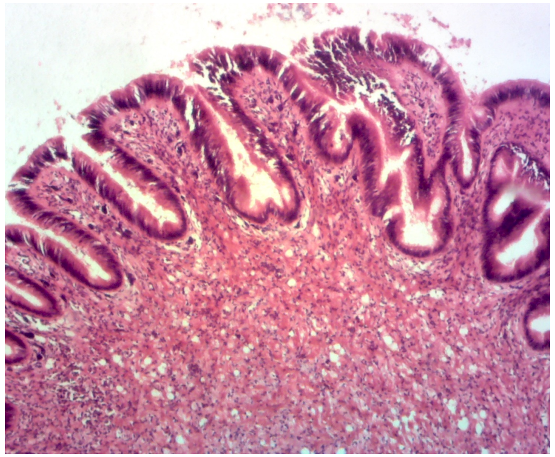

View 2

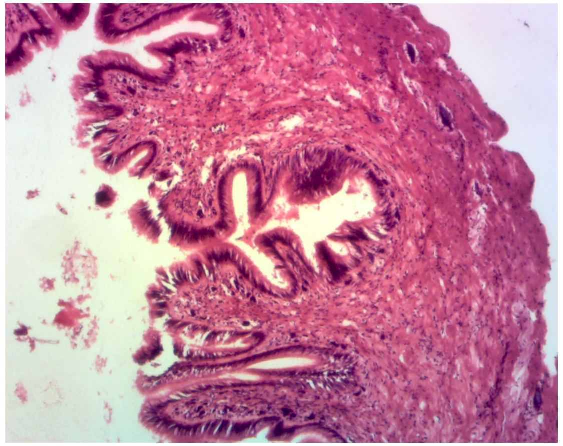

View 3
