# Supplementary material for: Impact of pesticides exposure on Archachatina marginata snails in four Cameroon monomodal rainforest sites
Source: PLoS One. 2024 Mar 4;19(3):e0297369. doi: 10.1371/journal.pone.0297369 (PMC10911591; doi:10.1371/journal.pone.0297369)
Supplement: Attachment — Submitted filename: Response to Reviewers .docx [file pone.0297369.s005.docx]

Enangue Njembele Annick Niquaise

IRAD/ Station specialized in Marine Ecosystem

PoBox 219 Kribi Cameroon

[enangueannick@gmail.com](mailto:enangueannick@gmail.com)

Kribi, 4^th^ November 2023

Object: Resubmission of manuscript

reference PONE-D-23-28780

To the Editor of the journal PLOS ONE

Dear Editor,

We are honored to hear that our manuscript entitled:” Impact of pesticides exposure on Archachatina marginata snails in four Cameroon monomodal rainforest sites” has a chance to be published in your prestigious journal. We are grateful for your and the reviewers’ comments and positive evaluation on our manuscript. Here is the response letter that address point by point all the comments and questions raised.

Please find enclosed with this letter, the revised version of manuscripts (the highlighted and the unmarked versions), the revised version of figures and tables files. We also attached the row data file.

The changes made are considerably improved the manuscript and do hope that the revised version is now suitable for publication. We look forward to hearing from you.

Your sincerely,

Annick Enangue Njembele, PhD

**Response to the editor and reviewers**

**Journal Requirements:**

1. **Please ensure that your manuscript meets PLOS ONE's style requirements, including those for file naming. The PLOS ONE style templates can be found at** [**https://journals.plos.org/plosone/s/file?id=wjVg/PLOSOne_formatting_sample_main_body.pdf**](https://journals.plos.org/plosone/s/file?id=wjVg/PLOSOne_formatting_sample_main_body.pdf) **and** [**https://journals.plos.org/plosone/s/fileid=ba62/PLOSOne_formatting_sample_title_authors_affiliations.pdf**](https://journals.plos.org/plosone/s/fileid=ba62/PLOSOne_formatting_sample_title_authors_affiliations.pdf)**.**

**Answer:** Changes were made accordingly

2. **We noted in your submission details that a portion of your manuscript may have been presented or published elsewhere. "These data were Presented in part at the 54th annual meeting of the Society for the Study of Reproduction, 15–18 December 2021, St. Louis, MO, USA". Please clarify whether this [conference proceeding or publication] was peer-reviewed and formally published. If this work was previously peer-reviewed and published, in the cover letter please provide the reason that this work does not constitute dual publication and should be included in the current manuscript.**

**Answer:** The portion of the manuscript was presented as an oral poster. There was not a peer reviewed process to present at that conference. However, the abstract appeared in the program of the conference. Also, the poster was uploaded on the website of the conference since it was a remote presentation done during the COVID period. More, the mass spectrometry result was not available at the time of the presentation.

3. **In your Data Availability statement, you have not specified where the minimal data set underlying the results described in your manuscript can be found. PLOS defines a study's minimal data set as the underlying data used to reach the conclusions drawn in the manuscript and any additional data required to replicate the reported study findings in their entirety. All PLOS journals require that the minimal data set be made fully available. For more information about our data policy, please see** [**http://journals.plos.org/plosone/s/data-availability**](http://journals.plos.org/plosone/s/data-availability)**.**Upon re-submitting your revised manuscript, please upload your study’s minimal underlying data set as either Supporting Information files or to a stable, public repository and include the relevant URLs, DOIs, or accession numbers within your revised cover letter. For a list of acceptable repositories, please see <http://journals.plos.org/plosone/s/data-availability#loc-recommended-repositories>. Any potentially identifying patient information must be fully anonymized.

**Answer:** Minimal data will be uploaded while submitting the reviewed manuscript.

4. **We note that Figure 1 in your submission contain [map/satellite] images which may be copyrighted. All PLOS content is published under the Creative Commons Attribution License (CC BY 4.0), which means that the manuscript, images, and Supporting Information files will be freely available online, and any third party is permitted to access, download, copy, distribute, and use these materials in any way, even commercially, with proper attribution. For these reasons, we cannot publish previously copyrighted maps or satellite images created using proprietary data, such as Google software (Google Maps, Street View, and Earth). For more information, see our copyright guidelines:** [**http://journals.plos.org/plosone/s/licenses-and-copyright**](http://journals.plos.org/plosone/s/licenses-and-copyright)**.**

**Answer:**  Figure 1 was modified Accordingly. There is no more need of copywriting permission. Actually, we now collaborated with a specialist in cartography that designed and provided a new version of the map. We also included that specialist as an author of this publication.

5. **Please note that tables (should remain/ be uploaded) as separate "table" files"**

**Answer:**  Modifications were made accordingly

**Reviewers' comments to the Author and Answers**

**Reviewer #1:**

**Study area: I recommend adding a description of sampling sites – for example - the main sources of pollution, industry, flow rates, population and others.**

**Answer:**  Modifications were made accordingly

**Results:** **The qualitative nature of the histological analysis. The histological analysis might have been more valuable to the reader if some indexes of severity were indicated. Currently, the scale is simply “positive” or “negative”. Some measure of severity would have helped.**

**Answer:**  Results are more explained in the reviewed manuscript by using the appropriate words of parts of the tissues that we were able to identify. The name was added in the pictures. For the kidney, we counted the number of vacuoles and did the comparison between every location via Welsh’s one-way ANOVA in order to point out the difference / index of severity between each collection sites.

**The figure statements are not labelled properly and do not provide enough detail. I should not have to look to the body of the results to figure out what these letters indicate. Figures and figure statements should stand alone.**

**Answer:**  Modifications were accordingly

**Reviewer #2:** 

**1-Please supply the journal with the GM data (row data)**

**Answer**: Row data were attached with the reviewed version of the manuscript

**2-Need to work on statistic and histology**

**Answer**:

**Statistic:** We compared the values of size and weight of animals collected from the different locations by using welsh’s one-way ANOVA. That statistic technique was used since the size of the samples were different from each location. To strengthen the result obtained, we compared two by two the values (size and weight) of locations with Student t-test. More, to have an overview of what is shown in the histograms of weight and size of snail, the picture of Fig 5 is showing from the bigger to the smaller snails of each collection.

**Histology:** For the quality of the picture, we uploaded corrected versions of histology sections microphotograph by using PACE as recommended by the journal. Results were as well more explained in the reviewed manuscript by using the appropriate words of parts of the tissues that we were able to identify. Names was added in the pictures. For the kidney, we counted the number of vacuoles and did the comparison between every location via Welsh’s one-way ANOVA in order to point out the difference between each collection sites. We also added a scale to better appreciate the sizes of animals.

**3- Add figure on the peak of the GM for the pesticides with the retention time**

**Answer**: While sending back the result of Mass spectrometry analysis, I was given 3 files that comprised concentrations of analyzed pesticides in the flesh, the method and the chromatogram showed in Fig 2. They did not provide the chromatogram with the name and the retention time of pesticides. When asked recently, the laboratory responded they do not have anymore that information since it was done in 2022. I even seek help to some chemists that were no able to identify each pesticides analyzed since the original file of the chromatogram was missing. Is it possible to consider only the concentrations showed in table 2?

**4- The point is not new**

**Answer**: Actually, this is the first paper showing the **presence of pesticides** in African giant **snails coming from the wild specifically from agricultural areas and where snails are often collected for consumption.** This result is showing that snails sold in the market in Cameroon might be pesticides-contaminated, since collection is the major way to supply them in the market. It is interesting since that could be a potential biohazard for human. Moreover, the result of this paper will have an impact in local population since it showed the pesticide contamination in natural condition. Consequently, that will increase the awareness on those community to not perform wild snails’ collection but in contrary, increase their rearing. More, we showed a disruption on two pesticides sensible organs (kidney and ovotestis). This result could imply that pesticides might affect their reproduction and detoxification. There are two other papers that showed the presence of pesticides and their impact but that was after exposure in controlled environment the impact was not observed on kidney which is a new information that we showed.

**5-How they did they send the sample to Egypt?**

**Answer**: Snails samples were dried for better conservation before sending by plane.

**6-The sequence and the plan of the paper are not clear**

**Answer**: The goal of this study was to evaluate the impact pesticides exposure of snail collected in the field on their general morphology, on two pesticides sensible organs (kidney and ovo-testis) and to the determine their pesticides presence on the flesh of these animal. The sequence used in the manuscript was structured to answer the following questions:

- First, are there any changes on snails’ general morphology due to pesticides exposure in the wild?
- Second, can we see disruption within some pesticides-sensible organs?
- Third, if there is any disruption, pesticides can them be found in animal flesh?

We modified the manuscript according to the above structure/sequence.

**7- The distribution of the histology is not enough and did not show any structure or the bar**

**Answer**: Actually, the quality of the staining can allow us to see properly the structure of analyzed organs. Concerning the scale bar, we uploaded the PACE recommended version that can allow to see the bar. Concerning the distribution, I showed contaminated in the same line vs non contaminated.

**8- I supposed to reject it but I gave them another chance**

**Answer**: We are grateful for the second chance to our paper. We made as best as possible all the changes requested.
