# Supplementary material for: Impact of pesticides exposure on Archachatina marginata snails in four Cameroon monomodal rainforest sites
Source: PLoS One. 2024 Mar 4;19(3):e0297369. doi: 10.1371/journal.pone.0297369 (PMC10911591; doi:10.1371/journal.pone.0297369)
Supplement: Peer review history

# Peer review: Decision Letter 0

**DOI:** 10.1371/journal.pone.0297369.r001

**Authors:** Selim Adewale Alarape

22 Sep 2023

PONE-D-23-28780Impact of pesticides exposure on Archachatina marginata snails in four Cameroon monomodal rainforest sitesPLOS ONE

Dear Dr. enangue njembele,

Thank you for submitting your manuscript to PLOS ONE. After careful consideration, we feel that it has merit but does not fully meet PLOS ONE’s publication criteria as it currently stands. Therefore, we invite you to submit a revised version of the manuscript that addresses the points raised during the review process.

ACADEMIC EDITOR: Be sure to:

- attend to the generality of the comments raised by the second reviewer who suggested major review of the manuscript
- Attention should also be focused on the comments as raised by the first reviewer

Please submit your revised manuscript by Nov 06 2023 11:59PM. If you will need more time than this to complete your revisions, please reply to this message or contact the journal office at plosone@plos.org. When you're ready to submit your revision, log on to https://www.editorialmanager.com/pone/ and select the 'Submissions Needing Revision' folder to locate your manuscript file.

Please include the following items when submitting your revised manuscript:

- A rebuttal letter that responds to each point raised by the academic editor and reviewer(s). You should upload this letter as a separate file labeled 'Response to Reviewers'.
- A marked-up copy of your manuscript that highlights changes made to the original version. You should upload this as a separate file labeled 'Revised Manuscript with Track Changes'.
- An unmarked version of your revised paper without tracked changes. You should upload this as a separate file labeled 'Manuscript'.

If you would like to make changes to your financial disclosure, please include your updated statement in your cover letter. Guidelines for resubmitting your figure files are available below the reviewer comments at the end of this letter.

If applicable, we recommend that you deposit your laboratory protocols in protocols.io to enhance the reproducibility of your results. Protocols.io assigns your protocol its own identifier (DOI) so that it can be cited independently in the future. For instructions see: https://journals.plos.org/plosone/s/submission-guidelines#loc-laboratory-protocols. Additionally, PLOS ONE offers an option for publishing peer-reviewed Lab Protocol articles, which describe protocols hosted on protocols.io. Read more information on sharing protocols at https://plos.org/protocols?utm_medium=editorial-email&utm_source=authorletters&utm_campaign=protocols.

We look forward to receiving your revised manuscript.

Kind regards,

Selim Adewale Alarape, MVPH

Academic Editor

PLOS ONE

Journal Requirements:

When submitting your revision, we need you to address these additional requirements.

1. Please ensure that your manuscript meets PLOS ONE's style requirements, including those for file naming. The PLOS ONE style templates can be found at https://journals.plos.org/plosone/s/file?id=wjVg/PLOSOne_formatting_sample_main_body.pdf and https://journals.plos.org/plosone/s/fileid=ba62/PLOSOne_formatting_sample_title_authors_affiliations.pdf.

2. We noted in your submission details that a portion of your manuscript may have been presented or published elsewhere. "These data were Presented in part at the 54th annual meeting of the Society for the Study of Reproduction, 15–18 December 2021, St. Louis, MO, USA".

Please clarify whether this [conference proceeding or publication] was peer-reviewed and formally published. If this work was previously peer-reviewed and published, in the cover letter please provide the reason that this work does not constitute dual publication and should be included in the current manuscript.

3. In your Data Availability statement, you have not specified where the minimal data set underlying the results described in your manuscript can be found. PLOS defines a study's minimal data set as the underlying data used to reach the conclusions drawn in the manuscript and any additional data required to replicate the reported study findings in their entirety. All PLOS journals require that the minimal data set be made fully available. For more information about our data policy, please see http://journals.plos.org/plosone/s/data-availability.

Upon re-submitting your revised manuscript, please upload your study’s minimal underlying data set as either Supporting Information files or to a stable, public repository and include the relevant URLs, DOIs, or accession numbers within your revised cover letter. For a list of acceptable repositories, please see http://journals.plos.org/plosone/s/data-availability#loc-recommended-repositories. Any potentially identifying patient information must be fully anonymized.

Important: If there are ethical or legal restrictions to sharing your data publicly, please explain these restrictions in detail. Please see our guidelines for more information on what we consider unacceptable restrictions to publicly sharing data: http://journals.plos.org/plosone/s/data-availability#loc-unacceptable-data-access-restrictions. Note that it is not acceptable for the authors to be the sole named individuals responsible for ensuring data access.

We will update your Data Availability statement to reflect the information you provide in your cover letter.

4. We note that Figure 1 in your submission contain [map/satellite] images which may be copyrighted. All PLOS content is published under the Creative Commons Attribution License (CC BY 4.0), which means that the manuscript, images, and Supporting Information files will be freely available online, and any third party is permitted to access, download, copy, distribute, and use these materials in any way, even commercially, with proper attribution. For these reasons, we cannot publish previously copyrighted maps or satellite images created using proprietary data, such as Google software (Google Maps, Street View, and Earth). For more information, see our copyright guidelines: http://journals.plos.org/plosone/s/licenses-and-copyright.

We require you to either (1) present written permission from the copyright holder to publish these figures specifically under the CC BY 4.0 license, or (2) remove the figures from your submission:

1. You may seek permission from the original copyright holder of Figure 1 to publish the content specifically under the CC BY 4.0 license.  

We recommend that you contact the original copyright holder with the Content Permission Form (http://journals.plos.org/plosone/s/file?id=7c09/content-permission-form.pdf) and the following text:

“I request permission for the open-access journal PLOS ONE to publish XXX under the Creative Commons Attribution License (CCAL) CC BY 4.0 (http://creativecommons.org/licenses/by/4.0/). Please be aware that this license allows unrestricted use and distribution, even commercially, by third parties. Please reply and provide explicit written permission to publish XXX under a CC BY license and complete the attached form.”

Please upload the completed Content Permission Form or other proof of granted permissions as an ""Other"" file with your submission.

In the figure caption of the copyrighted figure, please include the following text: “Reprinted from [ref] under a CC BY license, with permission from [name of publisher], original copyright [original copyright year].”

2. If you are unable to obtain permission from the original copyright holder to publish these figures under the CC BY 4.0 license or if the copyright holder’s requirements are incompatible with the CC BY 4.0 license, please either i) remove the figure or ii) supply a replacement figure that complies with the CC BY 4.0 license. Please check copyright information on all replacement figures and update the figure caption with source information. If applicable, please specify in the figure caption text when a figure is similar but not identical to the original image and is therefore for illustrative purposes only.

The following resources for replacing copyrighted map figures may be helpful:

USGS National Map Viewer (public domain): http://viewer.nationalmap.gov/viewer/

The Gateway to Astronaut Photography of Earth (public domain): http://eol.jsc.nasa.gov/sseop/clickmap/

Maps at the CIA (public domain): https://www.cia.gov/library/publications/the-world-factbook/index.html and https://www.cia.gov/library/publications/cia-maps-publications/index.html

NASA Earth Observatory (public domain): http://earthobservatory.nasa.gov/

Landsat: http://landsat.visibleearth.nasa.gov/

USGS EROS (Earth Resources Observatory and Science (EROS) Center) (public domain): http://eros.usgs.gov/#

Natural Earth (public domain): http://www.naturalearthdata.com/

5.  Please note that tables (should remain/ be uploaded) as separate "table" files"

[Note: HTML markup is below. Please do not edit.]

Reviewers' comments:

Reviewer's Responses to Questions

**Comments to the Author**

1. Is the manuscript technically sound, and do the data support the conclusions?

The manuscript must describe a technically sound piece of scientific research with data that supports the conclusions. Experiments must have been conducted rigorously, with appropriate controls, replication, and sample sizes. The conclusions must be drawn appropriately based on the data presented. 

Reviewer #1: Yes

Reviewer #2: No

**********

2. Has the statistical analysis been performed appropriately and rigorously? 

Reviewer #1: Yes

Reviewer #2: No

**********

3. Have the authors made all data underlying the findings in their manuscript fully available?

The PLOS Data policy requires authors to make all data underlying the findings described in their manuscript fully available without restriction, with rare exception (please refer to the Data Availability Statement in the manuscript PDF file). The data should be provided as part of the manuscript or its supporting information, or deposited to a public repository. For example, in addition to summary statistics, the data points behind means, medians and variance measures should be available. If there are restrictions on publicly sharing data—e.g. participant privacy or use of data from a third party—those must be specified.

Reviewer #1: Yes

Reviewer #2: No

**********

4. Is the manuscript presented in an intelligible fashion and written in standard English?

PLOS ONE does not copyedit accepted manuscripts, so the language in submitted articles must be clear, correct, and unambiguous. Any typographical or grammatical errors should be corrected at revision, so please note any specific errors here.

Reviewer #1: Yes

Reviewer #2: No

**********

5. Review Comments to the Author

Please use the space provided to explain your answers to the questions above. You may also include additional comments for the author, including concerns about dual publication, research ethics, or publication ethics. (Please upload your review as an attachment if it exceeds 20,000 characters)

Reviewer #1: Study area: I recommend adding a description of samplingsites – for example - the main sources of pollution, industry, flow rates, population and others.

Results: The qualitative nature of the histological analysis. The histological analysis might have been more valuable to the reader if some index of severity were indicated. Currently, the scale is simply “positive” or “negative”. Some measure of severity would have helped.

The figure statements are not labelled properly and do not provide enough detail. I should not have to look to the body of the results to figure out what these letters indicate. Figures and figure statements should stand alone.

Reviewer #2: all my comments in an attached file

please supply the journal with the GM data (row data)

need to work on statistic and histology

there are questions in the attached file , please answer them

add figure on the peak of the GM for the pesticides with the retention time

**********

6. PLOS authors have the option to publish the peer review history of their article (what does this mean?). If published, this will include your full peer review and any attached files.

If you choose “no”, your identity will remain anonymous but your review may still be made public.

**Do you want your identity to be public for this peer review?** For information about this choice, including consent withdrawal, please see our Privacy Policy.

Reviewer #1: No

Reviewer #2: No

**********

[NOTE: If reviewer comments were submitted as an attachment file, they will be attached to this email and accessible via the submission site. Please log into your account, locate the manuscript record, and check for the action link "View Attachments". If this link does not appear, there are no attachment files.]

While revising your submission, please upload your figure files to the Preflight Analysis and Conversion Engine (PACE) digital diagnostic tool, https://pacev2.apexcovantage.com/. PACE helps ensure that figures meet PLOS requirements. To use PACE, you must first register as a user. Registration is free. Then, login and navigate to the UPLOAD tab, where you will find detailed instructions on how to use the tool. If you encounter any issues or have any questions when using PACE, please email PLOS at figures@plos.org. Please note that Supporting Information files do not need this step.

# Author comment: Author response to Decision Letter 0

**DOI:** 10.1371/journal.pone.0297369.r002

11 Dec 2023

Enangue Njembele Annick Niquaise

IRAD/ Station specialized in Marine Ecosystem

PoBox 219 Kribi Cameroon

enangueannick@gmail.com

Kribi, 4th November 2023

Object: Resubmission of manuscript 

reference PONE-D-23-28780

 To the Editor of the journal PLOS ONE

Dear Editor,

We are honored to hear that our manuscript entitled:” Impact of pesticides exposure on Archachatina marginata snails in four Cameroon monomodal rainforest sites” has a chance to be published in your prestigious journal. We are grateful for your and the reviewers’ comments and positive evaluation on our manuscript. Here is the response letter that address point by point all the comments and questions raised. 

Please find enclosed with this letter, the revised version of manuscripts (the highlighted and the unmarked versions), the revised version of figures and tables files. We also attached the row data file.

The changes made are considerably improved the manuscript and do hope that the revised version is now suitable for publication. We look forward to hearing from you.

Your sincerely,

Annick Enangue Njembele, PhD

 

Response to the editor and reviewers

Journal Requirements:

1. Please ensure that your manuscript meets PLOS ONE's style requirements, including those for file naming. The PLOS ONE style templates can be found at https://journals.plos.org/plosone/s/file?id=wjVg/PLOSOne_formatting_sample_main_body.pdf and https://journals.plos.org/plosone/s/fileid=ba62/PLOSOne_formatting_sample_title_authors_affiliations.pdf.

Answer: Changes were made accordingly

2. We noted in your submission details that a portion of your manuscript may have been presented or published elsewhere. "These data were Presented in part at the 54th annual meeting of the Society for the Study of Reproduction, 15–18 December 2021, St. Louis, MO, USA". Please clarify whether this [conference proceeding or publication] was peer-reviewed and formally published. If this work was previously peer-reviewed and published, in the cover letter please provide the reason that this work does not constitute dual publication and should be included in the current manuscript.

Answer: The portion of the manuscript was presented as an oral poster. There was not a peer reviewed process to present at that conference. However, the abstract appeared in the program of the conference. Also, the poster was uploaded on the website of the conference since it was a remote presentation done during the COVID period. More, the mass spectrometry result was not available at the time of the presentation.

3. In your Data Availability statement, you have not specified where the minimal data set underlying the results described in your manuscript can be found. PLOS defines a study's minimal data set as the underlying data used to reach the conclusions drawn in the manuscript and any additional data required to replicate the reported study findings in their entirety. All PLOS journals require that the minimal data set be made fully available. For more information about our data policy, please see http://journals.plos.org/plosone/s/data-availability.Upon re-submitting your revised manuscript, please upload your study’s minimal underlying data set as either Supporting Information files or to a stable, public repository and include the relevant URLs, DOIs, or accession numbers within your revised cover letter. For a list of acceptable repositories, please see http://journals.plos.org/plosone/s/data-availability#loc-recommended-repositories. Any potentially identifying patient information must be fully anonymized.

Answer: Minimal data will be uploaded while submitting the reviewed manuscript.

4. We note that Figure 1 in your submission contain [map/satellite] images which may be copyrighted. All PLOS content is published under the Creative Commons Attribution License (CC BY 4.0), which means that the manuscript, images, and Supporting Information files will be freely available online, and any third party is permitted to access, download, copy, distribute, and use these materials in any way, even commercially, with proper attribution. For these reasons, we cannot publish previously copyrighted maps or satellite images created using proprietary data, such as Google software (Google Maps, Street View, and Earth). For more information, see our copyright guidelines: http://journals.plos.org/plosone/s/licenses-and-copyright.

Answer: Figure 1 was modified Accordingly. There is no more need of copywriting permission. Actually, we now collaborated with a specialist in cartography that designed and provided a new version of the map. We also included that specialist as an author of this publication. 

5. Please note that tables (should remain/ be uploaded) as separate "table" files"

Answer: Modifications were made accordingly

Reviewers' comments to the Author and Answers

Reviewer #1: 

Study area: I recommend adding a description of sampling sites – for example - the main sources of pollution, industry, flow rates, population and others.

Answer: Modifications were made accordingly

Results: The qualitative nature of the histological analysis. The histological analysis might have been more valuable to the reader if some indexes of severity were indicated. Currently, the scale is simply “positive” or “negative”. Some measure of severity would have helped.

Answer: Results are more explained in the reviewed manuscript by using the appropriate words of parts of the tissues that we were able to identify. The name was added in the pictures. For the kidney, we counted the number of vacuoles and did the comparison between every location via Welsh’s one-way ANOVA in order to point out the difference / index of severity between each collection sites.

The figure statements are not labelled properly and do not provide enough detail. I should not have to look to the body of the results to figure out what these letters indicate. Figures and figure statements should stand alone.

Answer: Modifications were accordingly

Reviewer #2: 

1-Please supply the journal with the GM data (row data)

Answer: Row data were attached with the reviewed version of the manuscript

2-Need to work on statistic and histology

Answer:

Statistic: We compared the values of size and weight of animals collected from the different locations by using welsh’s one-way ANOVA. That statistic technique was used since the size of the samples were different from each location. To strengthen the result obtained, we compared two by two the values (size and weight) of locations with Student t-test. More, to have an overview of what is shown in the histograms of weight and size of snail, the picture of Fig 5 is showing from the bigger to the smaller snails of each collection. 

Histology: For the quality of the picture, we uploaded corrected versions of histology sections microphotograph by using PACE as recommended by the journal. Results were as well more explained in the reviewed manuscript by using the appropriate words of parts of the tissues that we were able to identify. Names was added in the pictures. For the kidney, we counted the number of vacuoles and did the comparison between every location via Welsh’s one-way ANOVA in order to point out the difference between each collection sites. We also added a scale to better appreciate the sizes of animals.

3- Add figure on the peak of the GM for the pesticides with the retention time

Answer: While sending back the result of Mass spectrometry analysis, I was given 3 files that comprised concentrations of analyzed pesticides in the flesh, the method and the chromatogram showed in Fig 2. They did not provide the chromatogram with the name and the retention time of pesticides. When asked recently, the laboratory responded they do not have anymore that information since it was done in 2022. I even seek help to some chemists that were no able to identify each pesticides analyzed since the original file of the chromatogram was missing. Is it possible to consider only the concentrations showed in table 2?

4- The point is not new

Answer: Actually, this is the first paper showing the presence of pesticides in African giant snails coming from the wild specifically from agricultural areas and where snails are often collected for consumption. This result is showing that snails sold in the market in Cameroon might be pesticides-contaminated, since collection is the major way to supply them in the market. It is interesting since that could be a potential biohazard for human. Moreover, the result of this paper will have an impact in local population since it showed the pesticide contamination in natural condition. Consequently, that will increase the awareness on those community to not perform wild snails’ collection but in contrary, increase their rearing. More, we showed a disruption on two pesticides sensible organs (kidney and ovotestis). This result could imply that pesticides might affect their reproduction and detoxification. There are two other papers that showed the presence of pesticides and their impact but that was after exposure in controlled environment the impact was not observed on kidney which is a new information that we showed.

5-How they did they send the sample to Egypt?

Answer: Snails samples were dried for better conservation before sending by plane.

6-The sequence and the plan of the paper are not clear

Answer: The goal of this study was to evaluate the impact pesticides exposure of snail collected in the field on their general morphology, on two pesticides sensible organs (kidney and ovo-testis) and to the determine their pesticides presence on the flesh of these animal. The sequence used in the manuscript was structured to answer the following questions:

� First, are there any changes on snails’ general morphology due to pesticides exposure in the wild? 

� Second, can we see disruption within some pesticides-sensible organs? 

� Third, if there is any disruption, pesticides can them be found in animal flesh? 

We modified the manuscript according to the above structure/sequence.

7- The distribution of the histology is not enough and did not show any structure or the bar

Answer: Actually, the quality of the staining can allow us to see properly the structure of analyzed organs. Concerning the scale bar, we uploaded the PACE recommended version that can allow to see the bar. Concerning the distribution, I showed contaminated in the same line vs non contaminated.

8- I supposed to reject it but I gave them another chance

Answer: We are grateful for the second chance to our paper. We made as best as possible all the changes requested.

Attachment Submitted filename: Response to Reviewers .docx

# Editor report: Decision Letter 1

**DOI:** 10.1371/journal.pone.0297369.r003

**Authors:** Selim Adewale Alarape

4 Jan 2024

Impact of pesticides exposure on Archachatina marginata snails in four Cameroon monomodal rainforest sites

PONE-D-23-28780R1

Dear Dr. ANNICK NJEMBELE,

We’re pleased to inform you that your manuscript has been judged scientifically suitable for publication and will be formally accepted for publication once it meets all outstanding technical requirements.

Within one week, you’ll receive an e-mail detailing the required amendments. When these have been addressed, you’ll receive a formal acceptance letter and your manuscript will be scheduled for publication.

An invoice for payment will follow shortly after the formal acceptance. To ensure an efficient process, please log into Editorial Manager at http://www.editorialmanager.com/pone/, click the 'Update My Information' link at the top of the page, and double check that your user information is up-to-date. If you have any billing related questions, please contact our Author Billing department directly at authorbilling@plos.org.

If your institution or institutions have a press office, please notify them about your upcoming paper to help maximize its impact. If they’ll be preparing press materials, please inform our press team as soon as possible -- no later than 48 hours after receiving the formal acceptance. Your manuscript will remain under strict press embargo until 2 pm Eastern Time on the date of publication. For more information, please contact onepress@plos.org.

Kind regards,

Selim Adewale Alarape, MVPH

Academic Editor

PLOS ONE

Additional Editor Comments (optional):

Reviewers' comments:

# Editor report: Acceptance letter

**DOI:** 10.1371/journal.pone.0297369.r004

**Authors:** Selim Adewale Alarape

21 Feb 2024

PONE-D-23-28780R1 

PLOS ONE

Dear Dr. Enangue Njembele, 

I'm pleased to inform you that your manuscript has been deemed suitable for publication in PLOS ONE. Congratulations! Your manuscript is now being handed over to our production team.

At this stage, our production department will prepare your paper for publication. This includes ensuring the following:

* All references, tables, and figures are properly cited

* All relevant supporting information is included in the manuscript submission,

* There are no issues that prevent the paper from being properly typeset

If revisions are needed, the production department will contact you directly to resolve them. If no revisions are needed, you will receive an email when the publication date has been set. At this time, we do not offer pre-publication proofs to authors during production of the accepted work. Please keep in mind that we are working through a large volume of accepted articles, so please give us a few weeks to review your paper and let you know the next and final steps. 

Lastly, if your institution or institutions have a press office, please let them know about your upcoming paper now to help maximize its impact. If they'll be preparing press materials, please inform our press team within the next 48 hours. Your manuscript will remain under strict press embargo until 2 pm Eastern Time on the date of publication. For more information, please contact onepress@plos.org.

If we can help with anything else, please email us at customercare@plos.org.

Thank you for submitting your work to PLOS ONE and supporting open access. 

Kind regards, 

PLOS ONE Editorial Office Staff

on behalf of

Dr. Selim Adewale Alarape 

Academic Editor

PLOS ONE
